# Understanding different dominance patterns in western Amazonian forests

**DOI:** 10.1111/ele.14351

**Published:** 2023-12-18

**Authors:** Laura Matas‐Granados, Frederick C. Draper, Luis Cayuela, Julia G. de Aledo, Gabriel Arellano, Celina Ben Saadi, Timothy R. Baker, Oliver L. Phillips, Eurídice N. Honorio Coronado, Kalle Ruokolainen, Roosevelt García‐Villacorta, Katherine H. Roucoux, Maximilien Guèze, Elvis Valderrama Sandoval, Paul V. A. Fine, Carlos A. Amasifuen Guerra, Ricardo Zarate Gomez, Pablo R. Stevenson Diaz, Abel Monteagudo‐Mendoza, Rodolfo Vasquez Martinez, Jacob B. Socolar, Mathias Disney, Jhon del Aguila Pasquel, Gerardo Flores Llampazo, Jim Vega Arenas, José Reyna Huaymacari, Julio M. Grandez Rios, Manuel J. Macía

**Affiliations:** ^1^ Departamento de Biología, Área de Botánica Universidad Autónoma de Madrid Madrid Spain; ^2^ Centro de Investigación en Biodiversidad y Cambio Global (CIBC‐UAM) Universidad Autónoma de Madrid Madrid Spain; ^3^ School of Geography and Planning University of Liverpool Liverpool UK; ^4^ School of Geography University of Leeds Leeds UK; ^5^ Departamento de Biología y Geología, Física y Química Inorgánica Universidad Rey Juan Carlos, Móstoles Madrid Spain; ^6^ Ecology and Evolutionary Biology University of Michigan Ann Arbor Michigan USA; ^7^ Oikobit LLC, www.oikobit.com Albuquerque New Mexico USA; ^8^ School of Geography & Sustainable Development University of St Andrews St Andrews UK; ^9^ Department of Biology University of Turku Turku Finland; ^10^ Programa Restauración de Ecosistemas (PRE) Centro de Innovación Científica Amazónica (CINCIA) Puerto Maldonado Tambopata, Madre de Dios Peru; ^11^ Peruvian Center for Biodiversity and Conservation (PCBC) Iquitos Loreto Peru; ^12^ Man and Biosphere Programme UNESCO Paris Île‐de‐France France; ^13^ Facultad de Ciencias Biológicas Universidad Nacional de la Amazonía Peruana Iquitos Peru; ^14^ Department of Integrative Biology University of California Berkeley Berkeley California USA; ^15^ Escuela de Ingeniería Forestal, Facultad de Ingeniería y Ciencias Agrarias Universidad Nacional Toribio Rodríguez de Mendoza de Amazonas (UNTRM) Chachapoyas Peru; ^16^ Instituto de Investigaciones de la Amazonía Peruana Iquitos Peru; ^17^ Departamento de Ciencias Biológicas Universidad de los Andes Bogotá Colombia; ^18^ Universidad Nacional de San Antonio Abad del Cusco Cusco Peru; ^19^ Estación Biológica del Jardín Botánico de Missouri Oxapampa Peru; ^20^ NCX San Francisco California USA; ^21^ Department of Geography University College London London UK; ^22^ Universidad Nacional de la Amazonia Peruana Iquitos Peru

**Keywords:** abundance‐occupancy relationship, dispersal limitation, dominant species, ecological specialization, environmental filters, generalist, spatial aggregation, specialist, species competition, tropical tree communities

## Abstract

Dominance of neotropical tree communities by a few species is widely documented, but dominant trees show a variety of distributional patterns still poorly understood. Here, we used 503 forest inventory plots (93,719 individuals ≥2.5 cm diameter, 2609 species) to explore the relationships between local abundance, regional frequency and spatial aggregation of dominant species in four main habitat types in western Amazonia. Although the abundance‐occupancy relationship is positive for the full dataset, we found that among dominant Amazonian tree species, there is a strong negative relationship between local abundance and regional frequency and/or spatial aggregation across habitat types. Our findings suggest an ecological trade‐off whereby dominant species can be locally abundant (*local dominants*) or regionally widespread (*widespread dominants*), but rarely both (*oligarchs*). Given the importance of dominant species as drivers of diversity and ecosystem functioning, unravelling different dominance patterns is a research priority to direct conservation efforts in Amazonian forests.

## Graphical abstract

Dominance of neotropical tree communities by a few species is widely documented, but dominant trees show a variety of distributional patterns still poorly understood. Contrary to the widely supported positive abundance‐occupancy relationship in ecology, we found that among dominant Amazonian tree species, there is a strong negative relationship between local abundance and regional frequency and/or spatial aggregation across habitat types. Our findings suggest an ecological trade‐off whereby dominant species can be locally abundant (local dominants) or regionally widespread (widespread dominants), but rarely both (oligarchs).

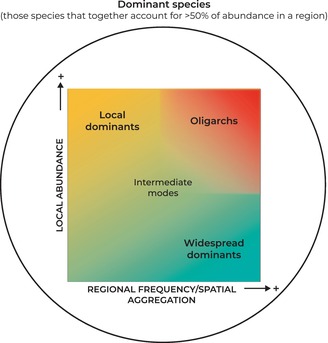

## PEER REVIEW

The peer review history for this article is available at https://www.webofscience.com/api/gateway/wos/peer‐review/10.1111/ele.14351.

## INTRODUCTION

In most ecological communities a few species are common while most are rare (Preston, 1948; Whittaker, 1965). This pattern holds regardless of whether one looks at numeric abundance, frequency and spatial extent (Gaston, 1994; Gaston et al., 2000). This ecological rule also applies in the most diverse ecosystems on Earth, such as Amazonian forests, where 2%–7% of tree species (‘*dominant species*’ from now on) account for 50% of individual trees (following ter Steege et al., 2013; but see also Pitman et al., 2001, 2013). Dominant species have a key role in driving large‐scale ecosystem functioning (Fauset et al., 2015) and spatial turnover in species composition (de Aledo et al., 2023; Draper et al., 2019). Therefore, changes in abundance and distribution of these dominant species will determine the response of tropical forests to global change in the coming decades (Avolio et al., 2019). A comprehensive understanding of the nature of dominance is essential to effectively respond to global change consequences.

Within Amazonian forests, numerous studies have documented patterns of dominance across different habitat types, forest strata and regions (Arellano & Macía, 2014; Draper et al., 2021; Macía, 2008, 2011; Macía & Svenning, 2005; Pitman et al., 2001). Nevertheless, how specifically dominant species balance regional frequency of occurrence and local abundance remains unknown. While some dominant tropical tree species form locally dense single‐species stands (Peh et al., 2011; ter Steege, Henkel, et al., 2019), others occur at relatively low densities in many plots and across large geographical areas (Pitman et al., 1999, 2001; ter Steege et al., 2013) and a few show high densities in a broad number of locations (i.e. oligarchic species) (Honorio Coronado et al., 2009; Macía, 2008, 2011; Pitman et al., 2001, 2013; ter Steege et al., 2013). Taken together, these studies may suggest that many different patterns of dominance exist and not all dominant species are characterized by both high abundances and frequencies. More widely across Earth's ecosystems, however, abundance, frequency and spatial extent are rarely independent from one another. Indeed, one of the most widely documented patterns in community ecology is a positive relationship between the local abundance and the regional frequency and/or spatial extent of a species—the abundance‐occupancy relationship (Gaston et al., 2000; He & Gaston, 2000b; Holt et al., 2002). Based on this general observed pattern, we would expect those species that have the highest local abundance to also occur in many sites and to have a large spatial extent.

Further, we may expect the relationship between local abundance and regional frequency to vary among habitat types due to variations in the proportion of specialist and generalist species. Within Amazonia, nutrient‐poor soil environments (e.g. white sand forests) tend to harbour a higher proportion of specialist taxa (Fine et al., 2010), while nutrient‐richer soil habitats (e.g. *terra firme* forests) have more generalist species (Duque et al., 2003; Pitman et al., 2001). There is no consensus on the relationship between local abundance, regional frequency and specialization (Denelle et al., 2020). Some hypothesize that specialists are more abundant in their optimal habitats than generalists because of the higher investment of resources needed by the latter to occupy several habitats (‘master‐of‐none’ hypothesis) (Levins, 1968; MacArthur, 1961). Conversely, others suggest that generalists perform better at any scale and, consequently, are likely to have both higher local abundances and regional frequencies (‘master‐of‐all’ hypothesis) (Brown, 1984). Therefore, the relative differences in specialists among habitat types may have a strong effect on the relationship between local abundance and regional frequency.

In this study, we used an extensive dataset consisting of 503 forest inventory plots across western Amazonia to explore different dominance patterns of tropical tree species in different habitat types (i.e. forest types) by quantifying their local abundance, regional frequency and spatial aggregation. This region is an ideal setting for this study because it is one of the most diverse areas of the Amazonian basin at both local and regional scales (Gentry, 1988; ter Steege et al., 2003; Wright, 2002) and potentially the most tree‐diverse region on the planet (Cazzolla Gatti et al., 2022; Sabatini et al., 2022). Furthermore, western Amazonia harbours different habitat types (Oliveira‐Filho et al., 2021), which have their own ecological and evolutionary processes which are responsible for changes in their floristic composition, species richness (Costa et al., 2020; Draper et al., 2018; Emilio et al., 2010; Tuomisto et al., 1995) and dominance (Pitman et al., 2001, 2014; Stropp et al., 2011). Specifically, we asked:

1. Is there a consistent relationship between local abundance and regional frequency among dominant tree species across western Amazonian forests? Here we test two alternative hypotheses:
  - Based on the well‐documented abundance‐occupancy relationship (Gaston et al., 2000), there will exist a positive relationship between local abundance and regional frequency.
  - Alternatively, based on previous work in Amazonian forests (Macía, 2008; Pitman et al., 2001; ter Steege et al., 2013; ter Steege, Henkel, et al., 2019), there will be a negative relationship between local abundance and regional frequency, whereby species can be either locally abundant or regionally frequent.
2. How does this relationship between local abundance and regional frequency vary among habitat types?
  - If a positive abundance‐frequency relationship emerges, we hypothesize that all habitat types will follow the same positive relationship following the empirical pattern found in previous work (Gaston, 1999; Gaston et al., 2000).
  - Alternatively, if a negative abundance‐frequency relationship exists, we hypothesize that in nutrient‐poor environments, which are characterized by drought and/or anoxic conditions (swamp and white sand forests) the relationship will be stronger compared with nutrient‐richer soil habitats (floodplain and *terra firme* forests), following the ‘master of none’ hypothesis (Levins, 1968; MacArthur, 1961).

Regional frequency is not always directly comparable to spatial aggregation when sampling plots are not uniformly distributed, as in this study, since dominant species with high regional frequency could be highly spatially aggregated by occurring in many closely‐situated plots. Therefore, we further explored spatial extent specifically asking:

3 Is there a consistent relationship between the spatial aggregation of dominant species and their regional frequency and local abundance across different western Amazonian forests?

For all habitat types, we hypothesize that:

- there will be a positive relationship between spatial aggregation and regional frequency, that is, dominant species that occur in few plots are more spatially aggregated than species occurring in many plots; and
- the relationship between local abundance and spatial aggregation will mirror the relationship between local abundance and regional frequency

## METHODS

### Floristic data and study area

**FIGURE 1:**
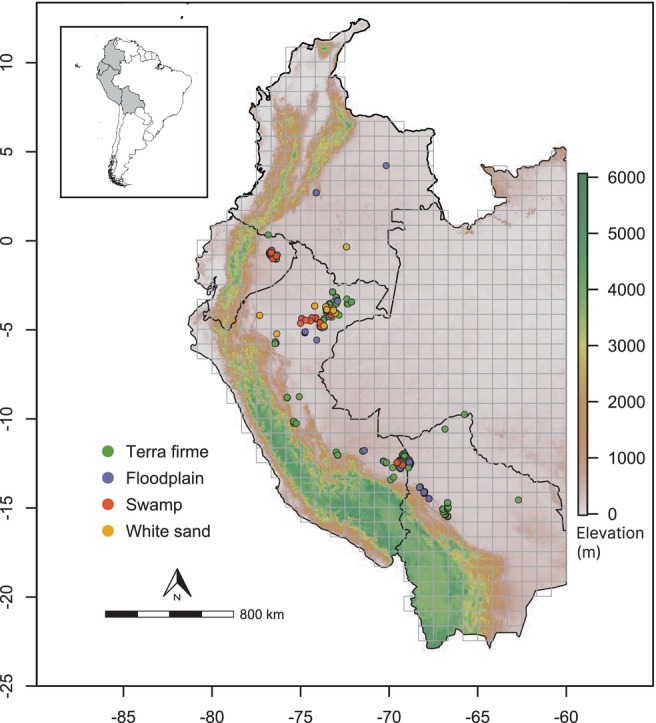
Map of the study sites in western Amazonia (Colombia, Ecuador, Peru and Bolivia) represented on a digital elevation model (Shuttle Radar Topography Mission [SRTM]) in WGS84 datum, latitude‐longitude coordinate reference system, including 100 × 100 km grid‐cells.

We used data from 503 forest inventory plots spread across western Amazonia, from Colombia to Bolivia (Figure 1). A total of 363 plots had an area of 0.1 ha, 134 plots were smaller than 0.1 ha (range from 0.025 to 0.08 ha), and 6 plots were larger (range from 0.128 to 0.213 ha). Plots are included in the RedGentry network (see Arellano et al., 2016; Draper et al., 2021 and Phillips et al., 2003 for details of sampling protocols). Many of these plots (55%) are curated and stored within ForestPlots.net (ForestPlots.net et al., 2021; Lopez‐Gonzalez et al., 2011). Across all plots, we measured stems with a diameter at breast height ≥2.5 cm within the plot limits (more details in metadata from the Dryad Digital Repository: https://doi.org/10.5061/dryad.pk0p2ngsd; Matas‐Granados et al., 2023).

Plots covered the main four habitat types in western Amazonia: 383 in *terra firme* (76%), 54 in floodplain (11%), 35 in swamp (7%) and 31 in white sand (6%) forests (see Appendix S1, Table S1 in Supplementary Information). *Terra firme* forests comprise the main habitat in western Amazonia, occupying up to 80% of the region (ter Steege et al., 2000). They are primarily defined by being never flooded and well drained (Condit et al., 2002; Fine & Kembel, 2011) and they usually have clay soils, which are relatively fertile by Amazonian standards (ter Steege et al., 2000). Floodplain forests are characterized by seasonal flooding events, being heterogenous both in terms of duration and level of flooding (Parolin et al., 2004), as well as in their soil nutrient content (de Assis et al., 2017). Swamp forests are characterized by poorly drained, permanently waterlogged and nutrient‐poor soils, which create an anoxic environment (Draper et al., 2018; Kahn, 1991; Pitman et al., 2014). They often occur as small island‐like patches in western Amazonia (Pitman et al., 2014; ter Steege et al., 2000), although they occupy much larger contiguous extensions in some parts of northeast Peru (Draper et al., 2014). White sand forests are scattered within *terra firme* landscapes, and occur on low pH oligotrophic sandy soils (Costa et al., 2020). All the studied habitats were located below 1000 m a.s.l. and they constituted a representative proportion of the study area, regarding their occurrence in western Amazonia (Stropp et al., 2011; ter Steege et al., 2000).

### Data processing

We excluded all individuals not identified to species level (mean 14% of individuals per plot), since plot data came from different projects and morphospecies were not cross‐checked. We also excluded individuals from doubtful identifications, e.g. ‘cf.’ and ‘aff.’ (mean 3% of individuals per plot). For the remaining individuals, we checked species names for synonyms and spelling mistakes, using the R package ‘Taxonstand’ (Cayuela et al., 2012). Identifications that were difficult to assign to a species were considered morphospecies and were also removed. Finally, we cross‐checked our species names list against the most recent checklists of Amazonian species (Cardoso et al., 2017; ter Steege, Mota de Oliveira, et al., 2019). Species not found in these checklists (635 species) were compared with collection records in the Tropicos database (Tropicos, 2019) and were excluded because: 572 species of them were growth forms not consistently included in all datasets (epiphytes, lianas, herbs and ferns), 25 were illegitimate Amazonian species with ranges outside of our region and 38 species were considered wrong identifications because they do not have recorded collection since their descriptions. After these filters, 2609 species and 93,719 individuals remained available for our analyses.

### Identifying dominant species

Since plot size varied among datasets, we identified dominant species as follows:

- We transformed the absolute abundances of each species into relative abundances following the formula to species *i* in plot *j*:
$$p_{\mathrm{ij}}=\frac{n_{\mathrm{ij}}}{n_{j}}$$
  where *n*_*ij*_ is the abundance of species *i* in plot *j*, and *n*_*j*_ is the total number of individuals in plot *j*.
- We calculated the accumulated relative abundance of each species adding the relative abundances of each species across all plots:
$$d_{i}=\sum_{j=1}^{\#\text{plots}}p_{\mathrm{ij}}$$
  This is the variable along which we ranked all species.
- The dichotomy between dominant and non‐dominant was based on the 50% threshold of $D=\sum_{i=1}^{\#\text{species}}d_{i}$ (Draper et al., 2019; ter Steege et al., 2013). We labelled as “dominant” those species that accumulated 50% of *D* when ranked from high to low *d*_*i*_.

We analysed separately dominant species by habitat type.

Since our plots are not evenly distributed in space, identifying dominant species considering all plots in each habitat type could favour the selection of spatially clumped species. To explore the effect of this potential bias, we divided our study area into equal 100 × 100 km squares (Figure 1), and we extracted 100 random subsamples from the complete set of plots in each habitat type drawing one plot from each square each time. We identified dominant species in the complete dataset and each subsample.

### Local abundance‐regional frequency relationship by habitat type

To test the relationship between local abundance and regional frequency of dominant species and the differences in the relationship across habitat types, we built beta regression models with a logit link function. We used the mean local relative abundance of each dominant species as the dependent variable (i.e. averaged across the plots where it occurred) and both the regional frequency (i.e. number of plots where a species occurred/total plots in the habitat type) and the habitat type (categorical) as predictors. We compared different alternative models using Akaike's information criterion (AIC), with the most complex model including the interaction between both predictors. Models with a difference in AIC >2 indicated that the worst model had no support and could be omitted. To fit models we used the *gam* function in the R package ‘gam’ (Hastie, 2022) and defined the beta error distribution with the *betar* function (as the family argument in ‘gam’) in the R package ‘mgcv’ (Wood, 2011). We conducted these analyses for: (i) the complete dataset, including all plots of each habitat type; and (ii) the 100 subsamples. We further wanted to explore how the tendency changed adding sequentially rarer species. Therefore, we conducted the same analyses for species that account for 60%, 70%, 80%, 85%, 90%, 92.5%, 95%, 97.5% and 100% of the total relative abundance.

We made graphs representing the relative abundance of each dominant species in each plot where it occurred (species‐level rank abundance distribution graphs) to explore changes in abundance at the single plot level. To do so, we proceeded as follows:

1. We calculated the four quartiles of mean local abundance and regional frequency of dominant species within each habitat type.
2. Based on the quartiles of the two variables, we classified dominant species at each habitat into 16 classes (i.e. all the combinations of the 4 quartiles of mean local abundance by the 4 quartiles of regional frequency).
3. Within each of these 16 classes, we visualized the rank abundance distribution of the dominant species belonging to that class.

### Spatial aggregation by habitat type

To study the spatial aggregation of species and their relationship with local abundance and regional frequency, we conducted two approaches:

First, we applied the negative binomial distribution (NBD) to quantify the strength of spatial aggregation of dominant species at the plot level. The *k* parameter of NBD makes reference to aggregation patterns: low values of *k* allude to more spatial aggregation while higher values result in less overdispersion, yielding the Poisson distribution (or “randomly distributed in space”) when *k* approaches infinity (Bliss & Fisher, 1953; He & Gaston, 2000a). Since NBD uses count data (number of individuals) and given the different plot sizes in our dataset, we estimated the number of individuals of each species in each plot considering the smallest plot surface within each habitat type (Table S1). We calculated the *k* parameter for all dominant species in each habitat type. Subsequently, we aimed to test the relationship of both local abundance and regional frequency of dominant species with their *k* parameter value. We built beta regression models with a logit link function separated into two groups: one with mean local abundance as the dependent variable and the other with regional frequency. All possible combinations between the *k* parameter value of dominant species and habitat type were used as predictors.

Second, the determination of *k* parameter is a measure of spatial aggregation at the plot scale, and it does not refer to the location of plots and how species are distributed at larger scales. Therefore, to study in more detail the spatial aggregation of the species at all scales of the study, we analysed the co‐dominance of each species at each spatial extent and habitat. In other words, we tested the probability of finding a conspecific of each species at each scale of the study area. To do so, we followed these steps:

1. For each species, we calculated the probability that two randomly chosen individuals in two plots (*j*, *k*) within the same habitat type belonged to the species *i* (Chave & Leigh, 2002; Leigh et al., 1993):
$$F_{i,\mathrm{jk}}=\frac{n_{\mathrm{ij}}\;n_{\mathrm{ik}}}{N_{j}N_{k}}$$

where *n*_*ij*_ is the number of individuals of the species *i* in the plot *j*; *n*_*ik*_, the number of individuals of the species *i* in the plot *k*; *Nj*, total number of individuals in plot *j*; and *N*_*k*_, total number of individuals in plot *k*.

2 We calculated the geographical distance between each pair of plots (*j*, *k*) within each habitat and related it to *F*_*i,jk*._ We smoothed and interpolated the data at missing geographical distances with a nonparametric regression estimator (*supsmu* function in the R package ‘stats’). By doing so, we estimated the curve of aggregation of each species at any given distance.
3 In general, species tend to be more aggregated at closer sites (a well‐known ecological pattern) and therefore, most curves described in (2) are monotonically decreasing (Figure S1). Visually comparing these distance‐decay curves of aggregation is difficult, especially at long distances, where *F* index values are very low for most species. To compare the differences in aggregation between species at each distance, we relativized the curve of aggregation of each species to the sum of all curves in each habitat type. Absolute and relative *F* values contain the same information (see Figure S1 for a visual comparison between the two approaches) but it is much easier to compare species when looking at the relative *F* values at each geographical distance. The curves of relative *F* estimate the probability of finding a conspecific of a species at each distance compared to the probability of finding conspecifics of any other species at that distance. As in the previous analysis, we represent the dominant species of each habitat type, grouped by the combination of quartiles of local abundance and regional frequency where they fell into.

All analyses were conducted in R v4.1.1 (R Core Team, 2022).

## RESULTS

### Identifying dominant species

From our complete dataset of 93,719 individuals belonging to 2609 species, we identified 106 dominant species in *terra firme* (4% of all species), 73 in floodplain (7%), 20 in swamp (5%) and 18 in white sand forests (4%) (Tables S1 and S6). All species found as dominant in the complete dataset were included as dominant in the list of dominant species gathered from the 100 subsamples (except *Inga ruiziana* in *terra firme* forests and *Dendropanax umbellatus* in white sand forests).

### Local abundance–regional frequency relationship by habitat type

**FIGURE 2:**
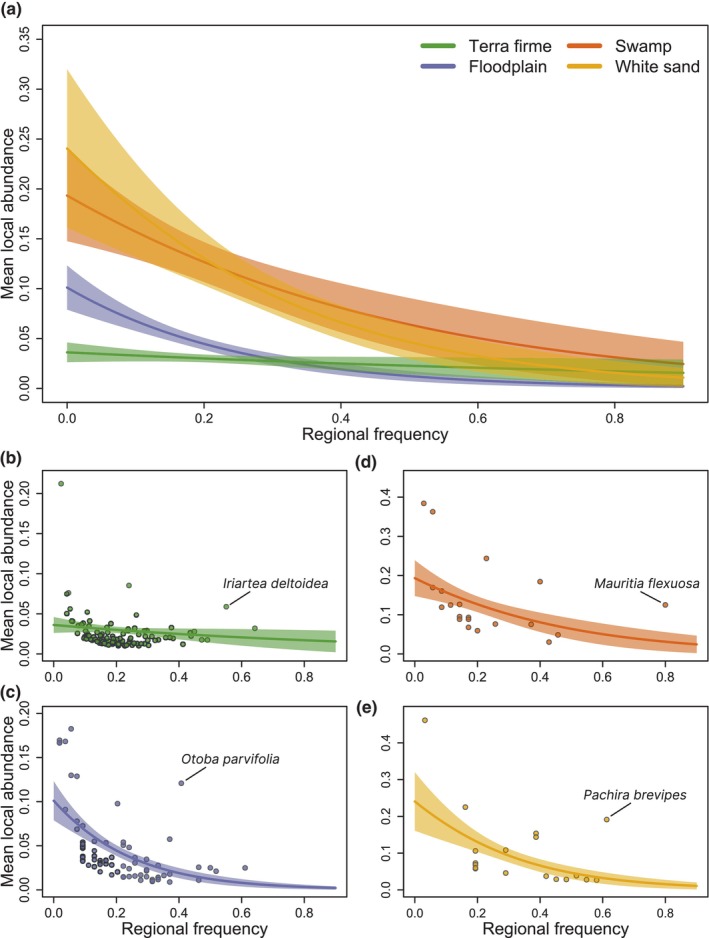
Model predictions for the best‐fit beta regression model showing the relationship between the mean local abundance and regional frequency of dominant species by habitat types. Lines represent mean generalized model fits, and shading represents 95% confidence intervals of model fits.

Both regional frequency and habitat type were relevant to predict the local abundance of dominant species (Tables S2 and S3). Regional frequency was negatively related to species’ local abundance in all habitat types: the more locally abundant they were, the less regionally frequent (Figure 2b–e). However, the relationship was more negative in white sand, followed by swamp, floodplain and *terra firme* forests (Figure 2a). Similar results were found for the 100 subsamples (Figure S2). However, there were exceptions to this rule, for example, *Iriartea deltoidea* in *terra firme*; *Otoba parvifolia* in floodplain; *Mauritia flexuosa* in swamp; and *Pachira brevipes* in white sand forests (Figure 2b–e). These four species were, on average, 95% regionally more frequent and 82% locally more abundant than the rest of the dominant species.

When we sequentially considered rarer species, we found that for the complete dataset, the tendency changed to positive when we considered species that account for 80% of the total relative abundance in *terra firme* forests, 97.5% in floodplains, 90% in swamps and 95% in white sands (Figures S3a and S4). These percentages of total relative abundance are accounted for 30% of total species in *terra firme* forests, 71% in floodplains, 41% in swamps and 50% in white sands (see results for the 100 subsamples, Figure S3b).

**FIGURE 3:**
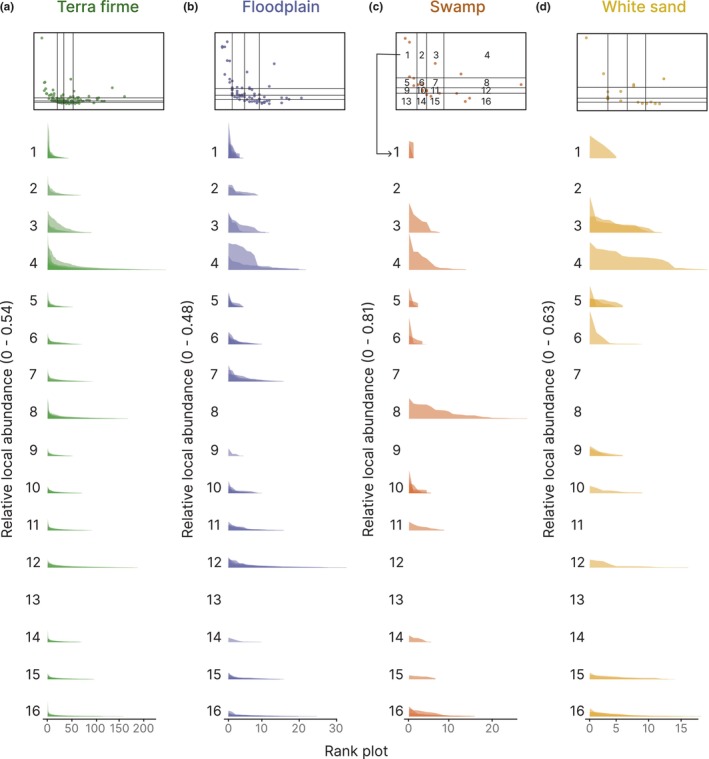
Species‐level rank abundance distribution graphs of dominant species by habitat type: (a) *terra firme*, (b) floodplain, (c) swamp and (d) white sand forests. Upper panels represent local abundance—regional frequency relationship of dominant species within each habitat type, with their quartiles. Numbers refer to each of the combination of quartiles of the two variables, local abundance and regional frequency.

When we analysed changes in abundance at the single plot level with rank abundance distribution curves, we found a similar pattern: generally dominant species that were regionally more frequent tended to be locally less abundant and, complementarily, dominant species that were locally more abundant tended to be regionally less frequent in all habitat types (Figure 3; similar results for 100 subsamples, Figure S5).

### Spatial aggregation by habitat type

All dominant species showed *k*‐parameter values ranging from 0 to 1 (Table S6). *k* parameter was negatively related to species local abundance and positively related to regional frequency across habitat types (Table S4, Figure S6, Table S5, Figure S7), with differences in their slopes among habitats (Figures S6a and S7a).

**FIGURE 4:**
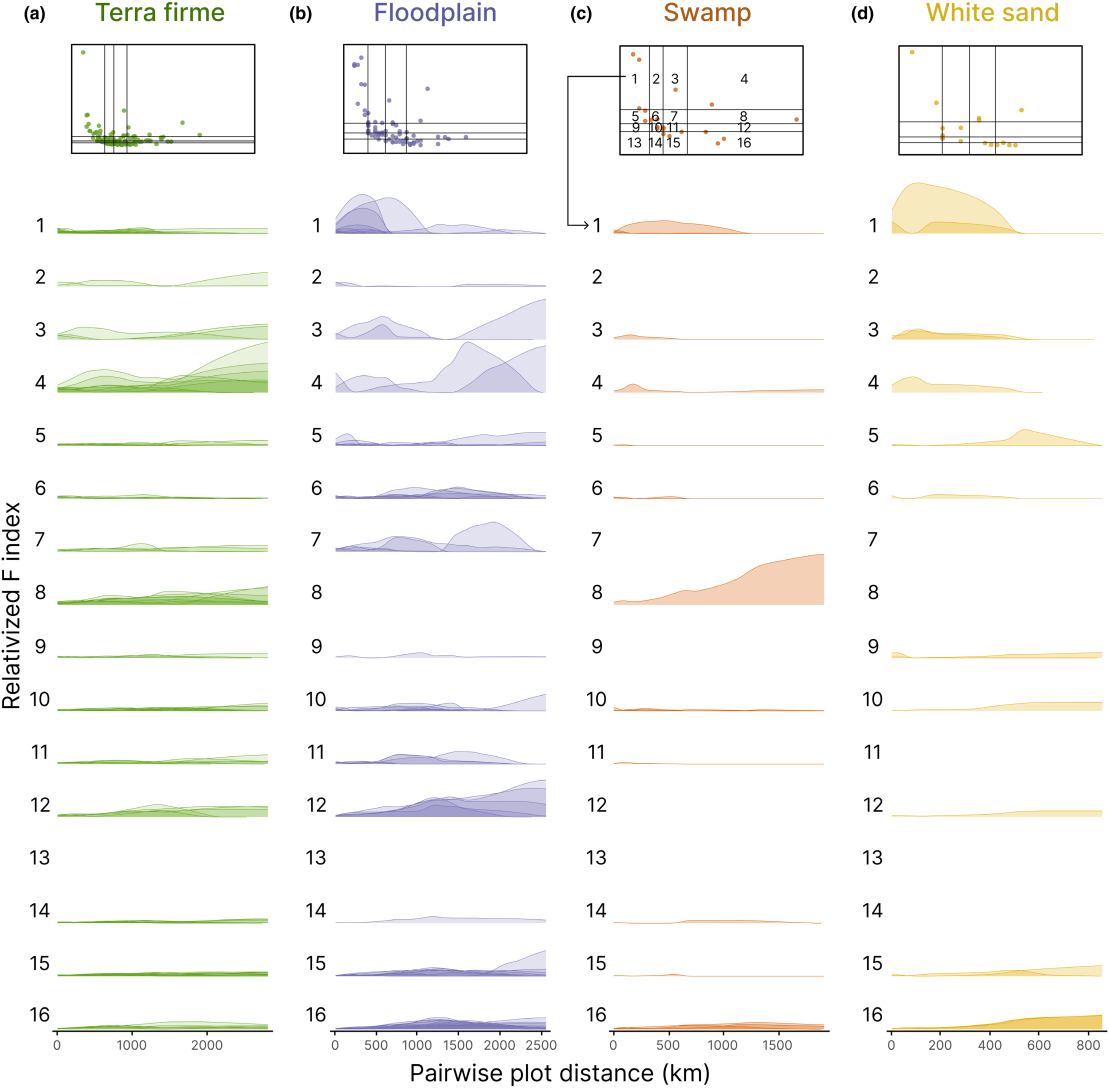
Relative spatial aggregation curves to dominant species by habitat type: (a) *terra firme*, (b) floodplain, (c) swamp and (d) white sand forests. Upper panels represent local abundance—regional frequency relationship of dominant species within each habitat type, with their quartiles. Numbers refer to each of the combination of quartiles of the two variables, local abundance and regional frequency. Each curve represents the probability of finding a conspecific of a species at each distance compared to the probability of finding conspecifics of any other species at that distance.

We also found differences in the spatial aggregation of dominant species at different spatial scales (Figure 4). In all habitats, the probability of finding a conspecific at short distances was relatively higher for dominant species that were locally more abundant but regionally less frequent. The complementary pattern was also true: the probability of finding a conspecific at long distances was relatively higher for dominant species that were locally less abundant but regionally more frequent (Figure 4). Oligarchic species (i.e. high local abundance and high regional frequency) were found to have a higher probability of finding a conspecific at any scale than most of the remaining dominant species, except for *Otoba parvifolia* in floodplain and *Pachira brevipes* in white sand forests (Cell 4 and 8; Figure 4a–d). We also found more noticeable differences in the spatial aggregation curves among dominant species in floodplain, swamp and white sand compared to *terra firme* forests (Figure 4).

## DISCUSSION

### Distinct dominance patterns in western Amazonian forests

**FIGURE 5:**
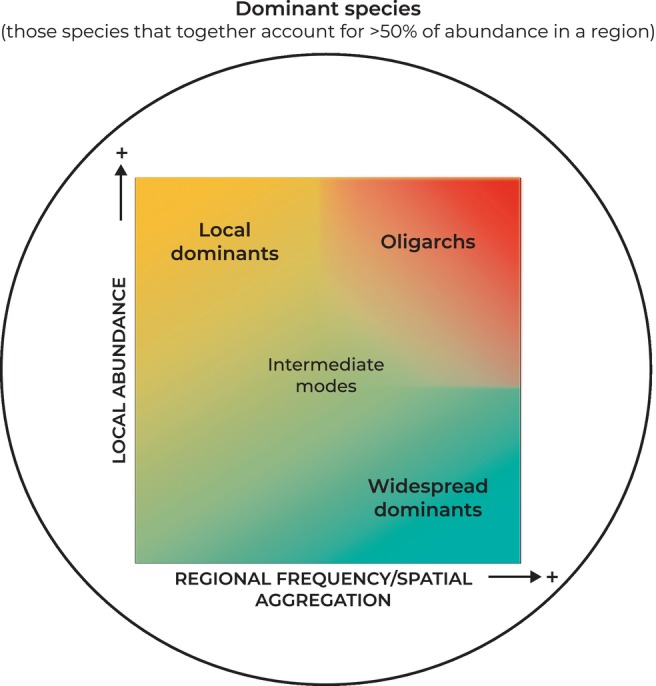
Conceptual framework of different dominance patterns inside the set of dominant species in four main habitat types of western Amazonia.

Our results show evidence of an ecological trade‐off whereby dominant species with high local abundance tend to occur in few locations and be spatially clustered, while dominant species that occur in many locations or have wide distributions tend to have relatively low local abundance. We propose three patterns of dominance, which, we suggest, are pervasive across Amazonia, and likely tropical forests more broadly: (1) *Local dominants*: species with high local abundance/low regional frequency and spatially aggregated; (2) *Widespread dominants*: species with low local abundance/high regional frequency and spatially dispersed; and (3) *Oligarchs*: species with both high local abundance/high regional frequency at any scale (Figure 5, some examples of each dominance pattern in Table S6).

Overall, there was a lower percentage of local dominants, some of which were found to have local abundances over 50% in previous research (ter Steege, Henkel, et al., 2019). Most dominant species tended to be widespread dominants, following previous work studying tree species distribution in Amazonian forests (Pitman et al., 1999) and only a few showed both high local abundance and regional frequency (i.e. oligarchs). Some of those species identified as oligarchs in our study were also previously reported as oligarchs in different western Amazonian habitats in previous research (Fine et al., 2010; Macía & Svenning, 2005; Pitman et al., 2001, 2014). Nevertheless, we emphasize that these categories represent extremes of a continuum of possibilities and most dominant species are typically found at intermediate levels (Figure 2).

Our findings appear to contrast with the widely supported positive abundance‐occupancy relationship, whereby locally abundant species are also regionally widespread (Gaston et al., 2000; Holt et al., 2002). The negative abundance‐occupancy relationship we found among Amazonian dominant species might be related to some factors: (1) the high environmental heterogeneity found in these habitat types, in terms of seasonality, rainfall and soil fertility, could favour ecological specialization of species (Dambros et al., 2020; Tuomisto et al., 2003), resulting in some dominant species occurring in few locations with specific suitable environmental conditions with high abundance but absent in other locations with different conditions. In relation to this potential factor, although our research did not focus on niche specialization per se, there are clear parallels between our local/widespread dominants and the specialist/generalist concepts. Specifically, we hypothesize that widespread dominants tend to be ecological generalists, while local dominants tend to be specialists. Our results therefore provide some support for the ‘master‐of‐none’ hypothesis (Levins, 1968; MacArthur, 1961), whereby generalists are more frequent but less locally abundant than specialist species, a pattern documented in other works with different taxa and regions (Denelle et al., 2020; Lawton, 1993; Verberk et al., 2010). (2) To a lesser extent, dispersal limitation, which is more restrictive in plants and more evident at regional scales, could avoid the colonization of available sites and, ultimately, restrict positive abundance‐occupancy relationships (Freckleton et al., 2005). This negative relationship appears to be more linked to the most regionally abundant species since the trend tend to change to positive when we sequentially included rarer species in our study (Figures S3 and S4; similar results in Fried et al., 2021), as found in previous works in tropical forests (Arellano et al., 2015; Macía & Svenning, 2005; Pitman et al., 2013).

### Different dominance patterns across habitat types

Our study also reveals the differences in the prevalence of each dominance pattern across western Amazonian forests:

In *terra firme* forests, the most dominant species tended to be widespread dominants. This pattern could be explained by three reasons: (1) the higher species richness of these forests (ter Steege et al., 2000) promotes high levels of local competition, preventing species to attain high local abundances (de Aledo et al., 2023); (2) a stronger effect of conspecific negative density dependence has been reported in species with acquisitive ecological strategies (Zang et al., 2021), which are more characteristic of *terra firme* forests than poorer soils environments (Fortunel et al., 2014). This phenomenon is likely to reduce recruitment of conspecific individuals at local scales, thereby preventing regionally dominant species from achieving high local abundances. (3) The vast contiguous extension covered by *terra firme* forests (ter Steege et al., 2000) promotes higher dispersal rates of species at regional scales (Dexter et al., 2017), facilitating dominant species to occur in more locations than those species that dominate in more isolated habitat types.

Floodplain forests appear to be an intermediary habitat type in terms of harbouring different dominance patterns. Their intrinsic intermediate conditions between *terra firme* and swamp and white sand forests can explain our findings: (1) their larger area compared to swamp and white sand forests in western Amazonia (ter Steege et al., 2000), along with riparian corridors (Wittmann et al., 2011), strengthens the connection among locations, promoting high dispersion and permitting species to occur in more locations (Parolin, 2009); (2) large area and high connectivity increases local species richness and, consequently, local competition, restricting high local abundances of species, such as in *terra firme* forests (de Aledo et al., 2023); (3) given the flooding variation across zones, some locations may have special environmental conditions that encourage the existence and adaptation of local dominants there (de Aledo et al., 2023; Parolin, 2009; Wittmann et al., 2006).

Finally, in nutrient‐poor soil habitats (swamp and white sand forests), our results showed a greater proportion of local dominants than in *terra firme* and, to a lesser extent, floodplain forests. These findings are consistent with other studies that show the ability to be locally abundant to be more prevalent in these habitats (Draper et al., 2018; ter Steege, Henkel, et al., 2019). Several reasons could explain this pattern: (1) strong environmental filters, such as permanent waterlogged and/or nutrient‐poor soils, significantly reduce species richness (Kotowski et al., 2010; Stropp et al., 2011), thereby reducing the number of competing species; (2) these habitat types are often characterized by a patchy reduced landscape distribution, with strong limitations on dispersal among patches at the landscape scale of western Amazonia (García‐Villacorta et al., 2016; Pitman et al., 2014). Therefore, even if a species is ideally suited to all patches of swamp forests, it may be unable to disperse to all locations. This assumption has some exceptions, such as *Mauritia flexuosa*, whose seeds are dispersed by mammals, humans and rivers (van der Hoek et al., 2019). (3) Furthermore, because some of these habitats (particularly peat swamps) are dynamic over centennial to millennial timescales (Draper et al., 2018), they are strongly affected by historical contingency and priority effects (Fukami, 2015). This implies that a species may have an advantage simply by occurring close by or arriving first to a location following a disturbance and achieving dominance before the arrival of others, perhaps better suited, competitors.

In summary, the prevalence of different dominance modes in each habitat type is probably influenced by both biotic and abiotic factors. Habitat availability and spatial connectivity among locations may enhance/restrict the regional occupancy/spatial aggregation of dominant species whereas species competition as well as environmental conditions and temporal dynamism may influence the local abundance of dominant species across habitat types.

### Future directions and practical implications

The results presented in our study may help to explain why no previous study has found any relationships between dominance and functional traits. If local dominance and widespread dominance are linked to different traits, it will be difficult to find clear relationships between dominance (in general) and functional traits. From this point of view, the range of trait values observed in dominant tree species is perhaps not surprising (Arellano et al., 2015; Fauset et al., 2015; ter Steege et al., 2013; ter Steege, Henkel, et al., 2019). We propose that different traits are linked to different dominance patterns, for example, we hypothesize that local dominant species will need to have traits that enable to deal with a high number of conspecific individuals and herbivory, i.e. a high investment in defence (Fine et al., 2006). Alternatively, we hypothesize that widespread dominant species will require traits that facilitate dispersal and will allocate fewer resources to defence, and more resources to growth and/or reproduction (Fine et al., 2006). Furthermore, since each habitat type shows different resource availability and different functional profiles in their species composition (Fortunel et al., 2012, 2014), the potential mechanisms underlying dominance patterns will be different across habitat types. We believe that testing these hypotheses associated with distinct functional strategies and habitat types represents a logical next step in the study of dominance in tropical forests.

Finally, our findings may help to better understand and predict the response of Amazonian forests to global change, because population changes in species with different dominance patterns may lead to profound differences in overall ecosystem responses (Avolio et al., 2019). More specifically, if population sizes of some local dominants are significantly reduced through species‐specific increases in mortality, this could have important consequences for the structure and function of regions within each habitat type. Alternatively, even dramatic reductions of some widespread dominants may have little overall effect on ecosystem functioning as it is more likely that different species would be able to occupy the space left behind. Eventually, the loss of oligarchs may result in even more drastic consequences. For example, the increase in mortality of the oligarchic palm *Mauritia flexuosa*, which is already happening due to destructive harvests in some regions of Peruvian Amazon, could result in the transformation of the structure and functioning of vast tracts of palm swamp forests (Endress et al., 2013). We thereby advocate for greater recognition of the importance of different dominance patterns and a more focused assessment of species‐specific responses of Amazonian trees to global change.

## AUTHOR CONTRIBUTIONS

Laura Matas‐Granados, Frederick C. Draper and Manuel J. Macía conceived the ideas. Laura Matas‐Granados and Frederick C. Draper designed the study with input from Luis Cayuela, Julia G. de Aledo and Manuel J. Macía. Laura Matas‐Granados performed the analyses with substantial input from Frederick C. Draper, Luis Cayuela, Gabriel Arellano and Jacob B. Socolar. Laura Matas‐Granados and Frederick C. Draper led the writing with substantial input from Luis Cayuela, Julia G. de Aledo, Gabriel Arellano, Celina Ben Saadi, Timothy R. Baker, Oliver L. Phillips, Eurídice N. Honorio Coronado, Kalle Ruokolainen, Roosevelt García‐Villacorta, Katherine H. Roucoux and Manuel J. Macía. All other coauthors contributed data and had the opportunity to comment on the manuscript.

## CONFLICT OF INTEREST STATEMENT

The authors have no conflicts of interest to declare.

## Supporting information


Data S1.

## Data Availability

Data and code are provided from the Dryad Digital Repository (https://doi.org/10.5061/dryad.pk0p2ngsd), Zenodo (https://doi.org/10.5281/zenodo.10143366) and ForestPlots (https://doi.org/10.5521/forestplots.net/2023_4).
