# Supplementary material for: Understanding different dominance patterns in western Amazonian forests
Source: Ecol Lett. 2023 Dec 18;27(1):e14351. doi: 10.1111/ele.14351 (PMC10952671; doi:10.1111/ele.14351)
Supplement: Supplementary file 1 — Data S1. [file ELE-27-0-s001.docx]

**SUPPLEMENTARY INFORMATION**

Understanding different dominance patterns in western Amazonian forests

Laura Matas-Granados, Frederick C. Draper, Luis Cayuela, Julia G. de Aledo, Gabriel Arellano, Celina Ben Saadi, Timothy R. Baker, Oliver L. Phillips, Eurídice N. Honorio Coronado, Kalle Ruokolainen, Roosevelt García-Villacorta, Katherine H. Roucoux, Maximilien Guèze, Elvis Valderrama Sandoval, Paul V.A. Fine, Carlos A. Amasifuen Guerra, Ricardo Zarate Gomez, Pablo R. Stevenson Diaz, Abel Monteagudo-Mendoza, Rodolfo Vasquez Martinez, Jacob B. Socolar, Mathias Disney, Jhon del Aguila Pasquel, Gerardo Flores Llampazo, Jim Vega Arenas, José Reyna Huaymacari, Julio M. Grandez Rios, Manuel J. Macía

**APPENDIX S1: SUPPORTIVE STATISTICAL INFORMATION**

**Table S1.**  Summary of forest inventory plot data used in this study.

| **Plot attributes** | ***Terra firme* forests** | **Floodplain forests** | **Swamp forests** | **White-sand forests** |
| --- | --- | --- | --- | --- |
| No. of plots | 383 | 54 | 35 | 31 |
| Plot size | 0.04 – 0.213 | 0.04 – 0.133 | 0.04 – 0.1 | 0.025 – 0.1 |
| Total stems | 69,628 | 10,644 | 6,225 | 7,222 |
| Median stems by plot | 182 | 188 | 115 | 211 |
| Maximum stems by plot | 813 | 1,002 | 874 | 573 |
| Minimum stems by plot | 20 | 20 | 21 | 26 |
| Total species | 2,374 | 1,091 | 434 | 442 |
| No. of dominant species from the complete dataset | 106 | 73 | 20 | 18 |
| Mean no. of dominant species from the 100 subsamples | 90 | 59 | 15 | 14 |
| Min no. of dominant species from the 100 subsamples | 76 | 43 | 8 | 8 |
| Max no. of dominant species from the 100 subsamples | 101 | 77 | 22 | 20 |

**Table S2.** Summary of beta regression models with mean local abundance against regional frequency of dominant species and habitat type from the complete dataset. “Deviance” refers to explained deviance (D^2^) as a measure of goodness of fit (Guisan & Zimmermann 2000). df = degrees of freedom; AIC = Akaike’s information criterion. The best-fit model is highlighted in bold.

| Model | df | AIC | Deviance |
| --- | --- | --- | --- |
| No explanatory variables | 2 | -864.324 | 0.001 |
| Mean local abundance ~ Regional frequency | 3 | -871.874 | 0.047 |
| Mean local abundance ~ Habitat type | 5 | -944.979 | 0.361 |
| Mean local abundance ~ Regional frequency + Habitat type | 6 | -995.449 | 0.519 |
| **Mean local abundance ~ Regional frequency * Habitat type** | **9** | **-1000.130** | **0.545** |

**Table S3.** Summary of the best beta regression model with “Mean local abundance” as the response variable. Statistically significant terms (p-value ≤ 0.05) are highlighted in bold.

| Term | Estimate | Std. error | z value | p-value |
| --- | --- | --- | --- | --- |
| **Intercept** | **-2.1851** | **0.1243** | **- 17.586** | **<0.001** |
| **Regional frequency** | **- 4.3884** | **0.6073** | **- 7.226** | **<0.001** |
| **Habitat type (Swamp)** | **0.7565** | **0.1944** | **3.892** | **<0.001** |
| **Habitat type (*Terra firme*)** | **- 1.0980** | **0.1908** | **- 5.755** | **<0.001** |
| **Habitat type (White sand)** | **1.0353** | **0.2544** | **4.070** | **<0.001** |
| **Regional frequency:Swamp** | **1.8786** | **0.8904** | **2.110** | **0.035** |
| **Regional frequency:*Terra firme*** | **3.4305** | **0.8756** | **3.918** | **<0.001** |
| Regional frequency:White sand | 0.6445 | 0.9319 | 0.692 | 0.489 |

**Table S4.** Summary of beta regression models with mean local abundance against k parameter values of dominant species and habitat type. “Deviance” refers to explained deviance (D^2^) as a measure of goodness of fit (Guisan & Zimmermann 2000). df = degrees of freedom; AIC = Akaike’s information criterion. The best-fit models are highlighted in bold.

| Model | df | AIC | Deviance |
| --- | --- | --- | --- |
| No explanatory variables | 2 | -858.921 | 0.001 |
| Mean local abundance ~ k parameter | 5 | -897.037 | 0.185 |
| Mean local abundance ~ Habitat type | 3 | -939.891 | 0.363 |
| Mean local abundance ~ k parameter + Habitat type | 6 | -995.123 | 0.533 |
| **Mean local abundance ~ k parameter * Habitat type** | **9** | **-998.401** | **0.555** |

**Table S5.** Summary of beta regression models with frequency against k parameter values of dominant species and habitat type. “Deviance” refers to explained deviance (D^2^) as a measure of goodness of fit (Guisan & Zimmermann 2000). df = degrees of freedom; AIC = Akaike’s information criterion. The best-fit model is highlighted in bold.

| Model | df | AIC | Deviance |
| --- | --- | --- | --- |
| No explanatory variables | 2 | -296.844 | 0.000 |
| Regional frequency ~ k parameter | 5 | -302.850 | 0.494 |
| Regional frequency ~ Habitat type | 3 | -432.027 | 0.058 |
| Regional frequency ~ k parameter + Habitat type | 6 | -463.891 | 0.580 |
| **Regional frequency ~ k parameter * Habitat type** | **9** | **-480.024** | **0.624** |


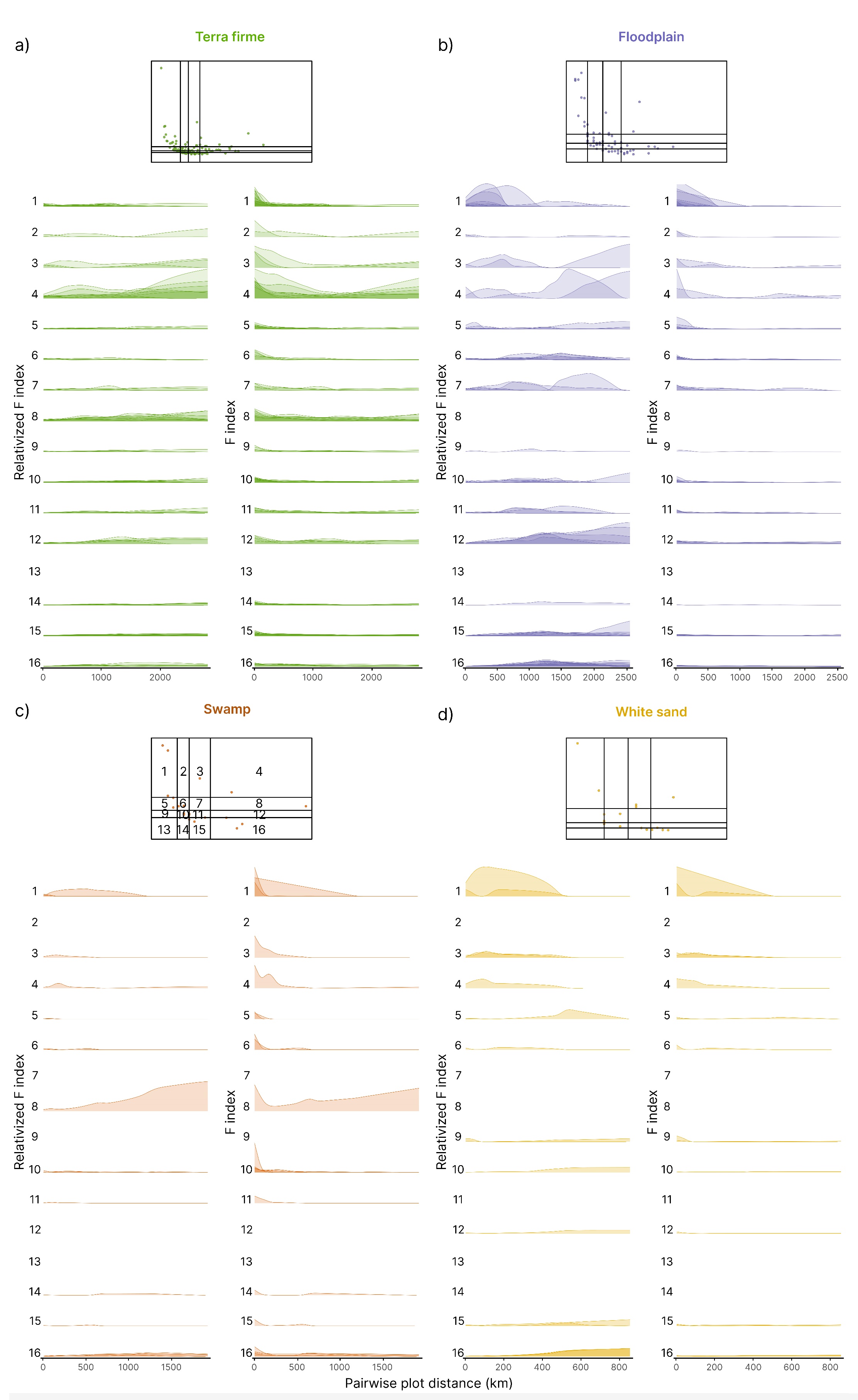


Figure S1. Visual comparisons of curves of spatial aggregation of dominant species across pairwise plot distances with absolute and relative F values by habitat type: a) *terra firme*, b) floodplain, c) swamp and d) white sand forests. Upper panels represent local abundance-regional frequency relationship of dominant species within each habitat type, with their quartiles. Numbers refer to each of the combination of quartiles of the two variables, local abundance and regional frequency.


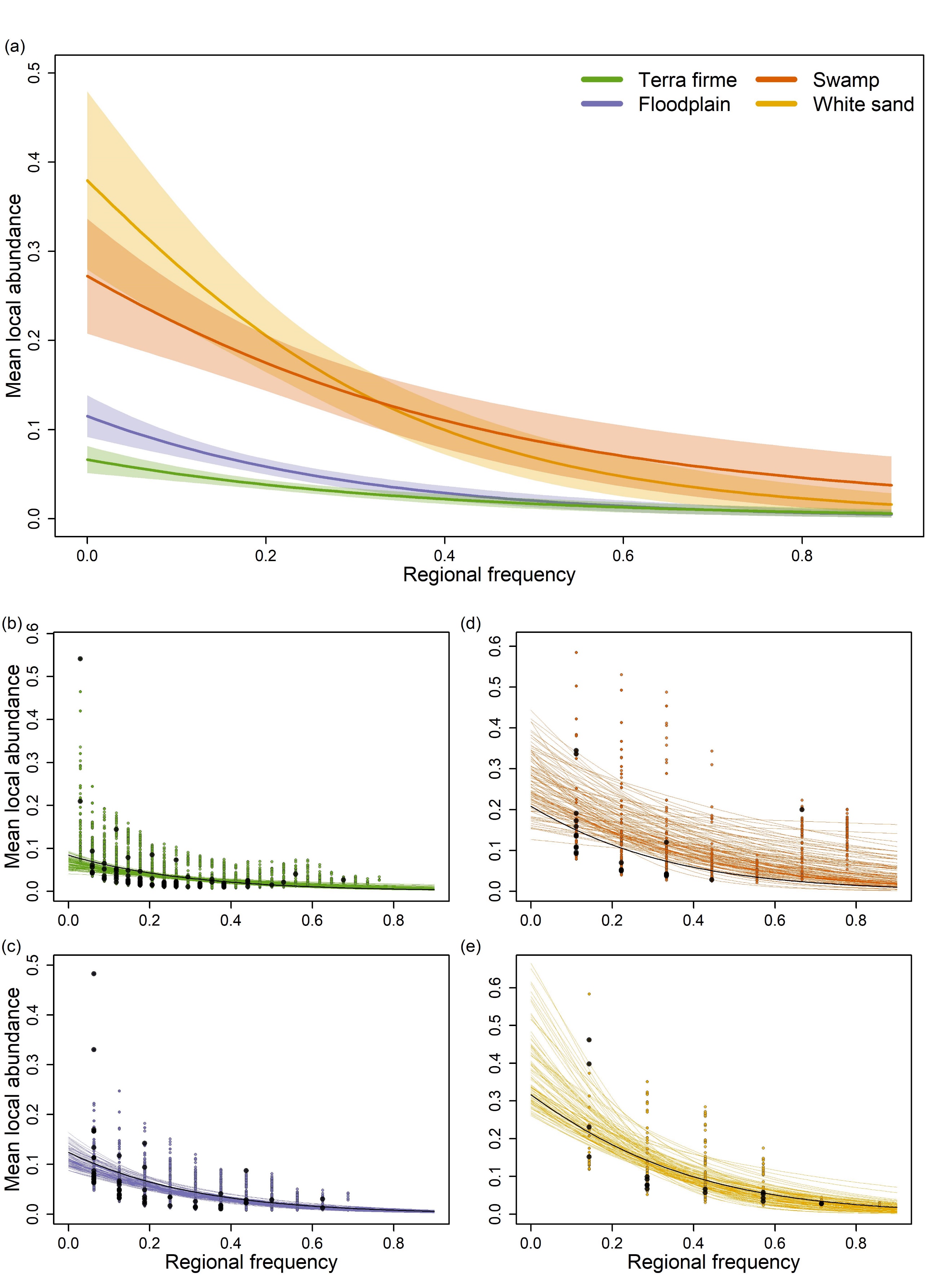


Figure S2. Model predictions for the best-fit beta regression model of 100 subsamples drawing one plot per 100 x 100 km square showing the relationship between the mean local abundance and the regional frequency of dominant species by habitat types. In (a) lines represent mean of the 100 generalized model fits, and shading represents 95% confidence intervals of the 100 model fits. In (b, c, d, e) we highlighted in black the results for 1 from 100 subsamples.


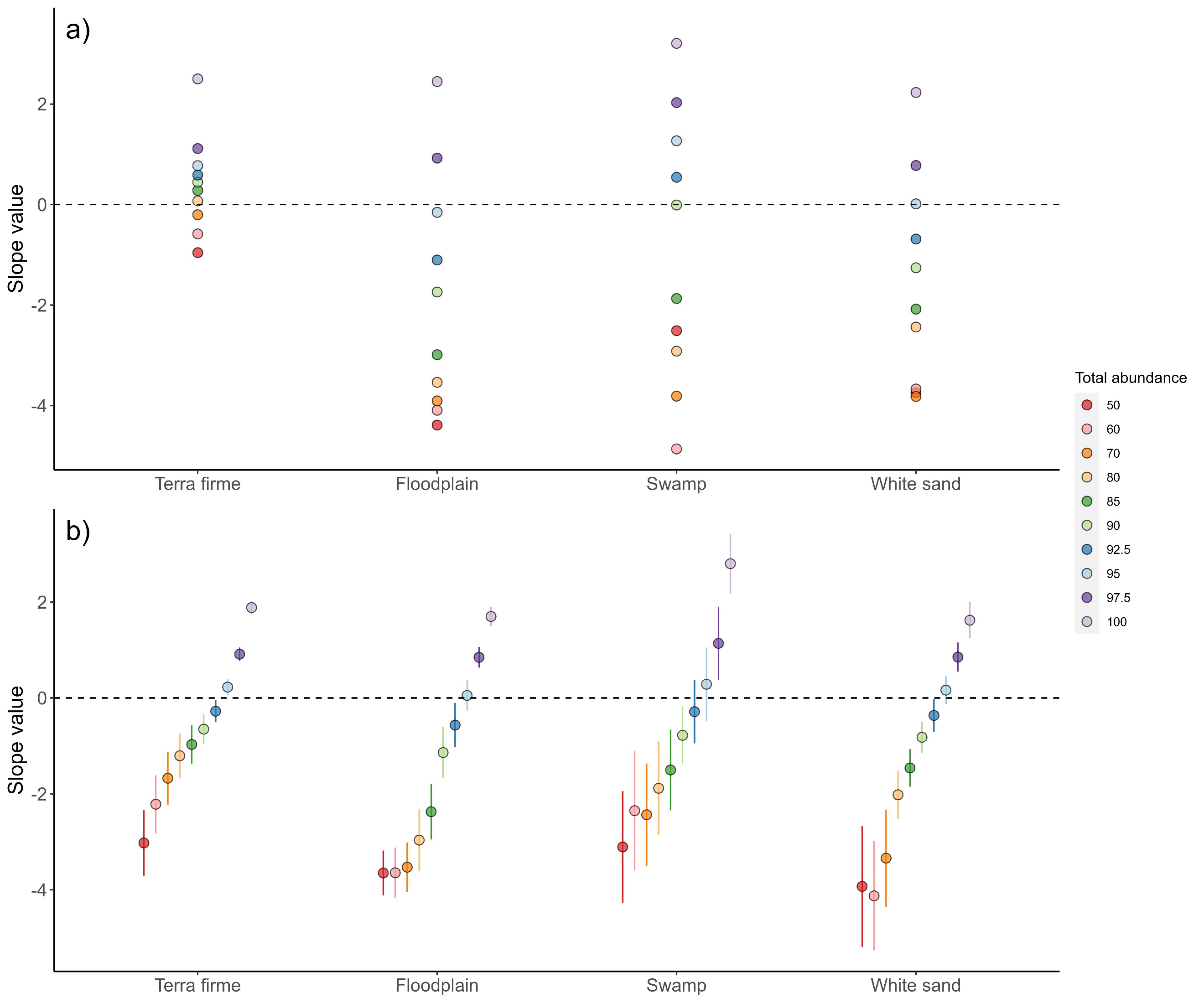


Figure S3. Slope values for the best-fit beta regression model showing the relationship between the mean local abundance and regional frequency of species by habitat type for a) the complete dataset and b) for 100 subsamples drawing one plot by 100 x 100 km square of the study area. We ran the abundance-frequency analysis including sequentially species that account for 60%, 70%, 80%, 85%, 90%, 92.5%, 95%, 97.5% and 100% of total relative abundance (showed as “Total abundance”). In b) points represent the median of the 100 subsamples of each level and vertical lines the standard deviations.


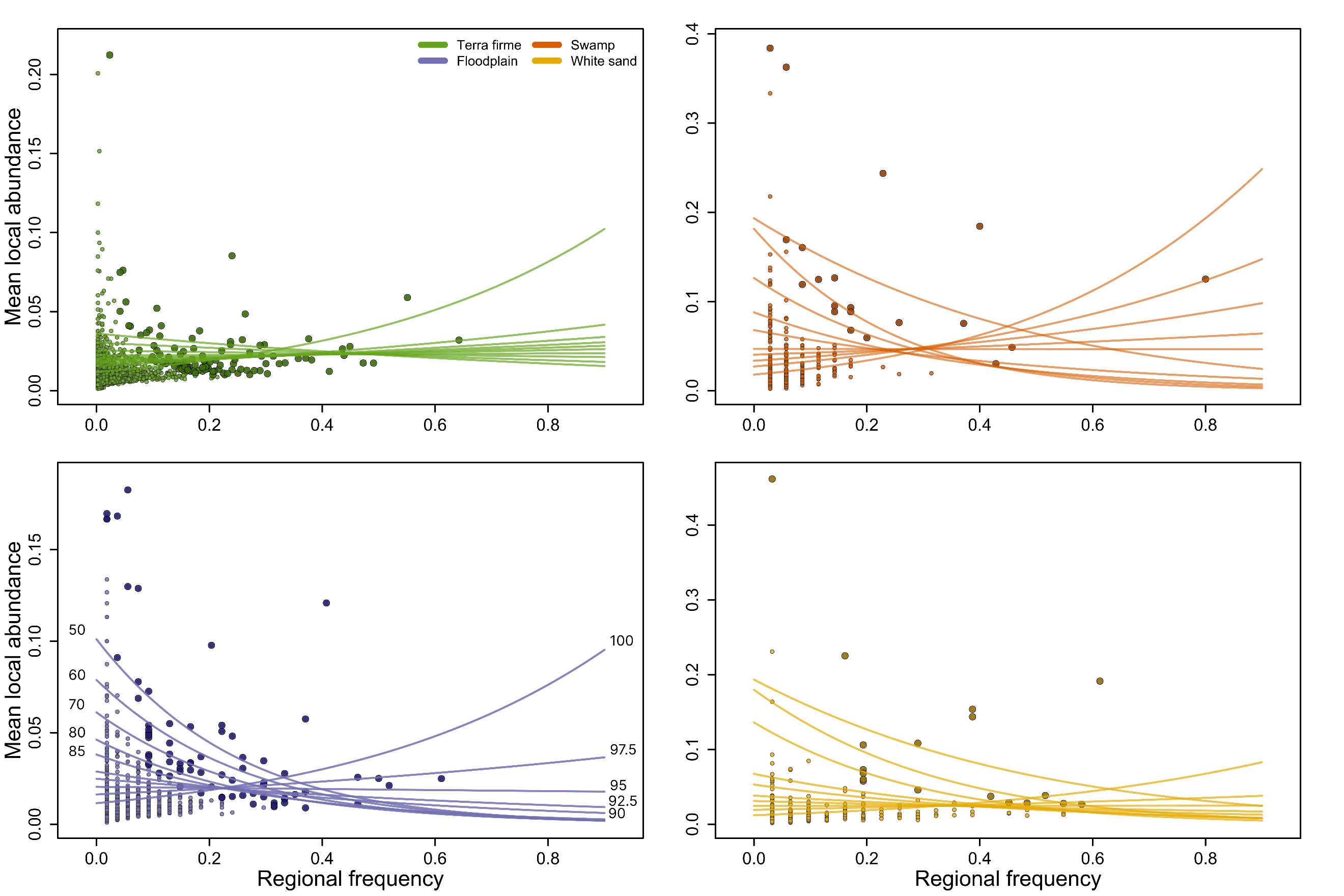


Figure S4. Model predictions for the best-fit beta regression model showing the relationship between the mean local abundance and the regional frequency of dominant species (50), species that account for 60% of the total relative abundance (60), 70% (70), 80% (80), 85% (85), 90% (90), 92.5% (92.5), 95% (95), 97.5% (97.5), 100% (100) by habitat types. Lines represent mean generalized model fits, points represent all species by habitat type and highlighted points represent dominant species of each habitat type.


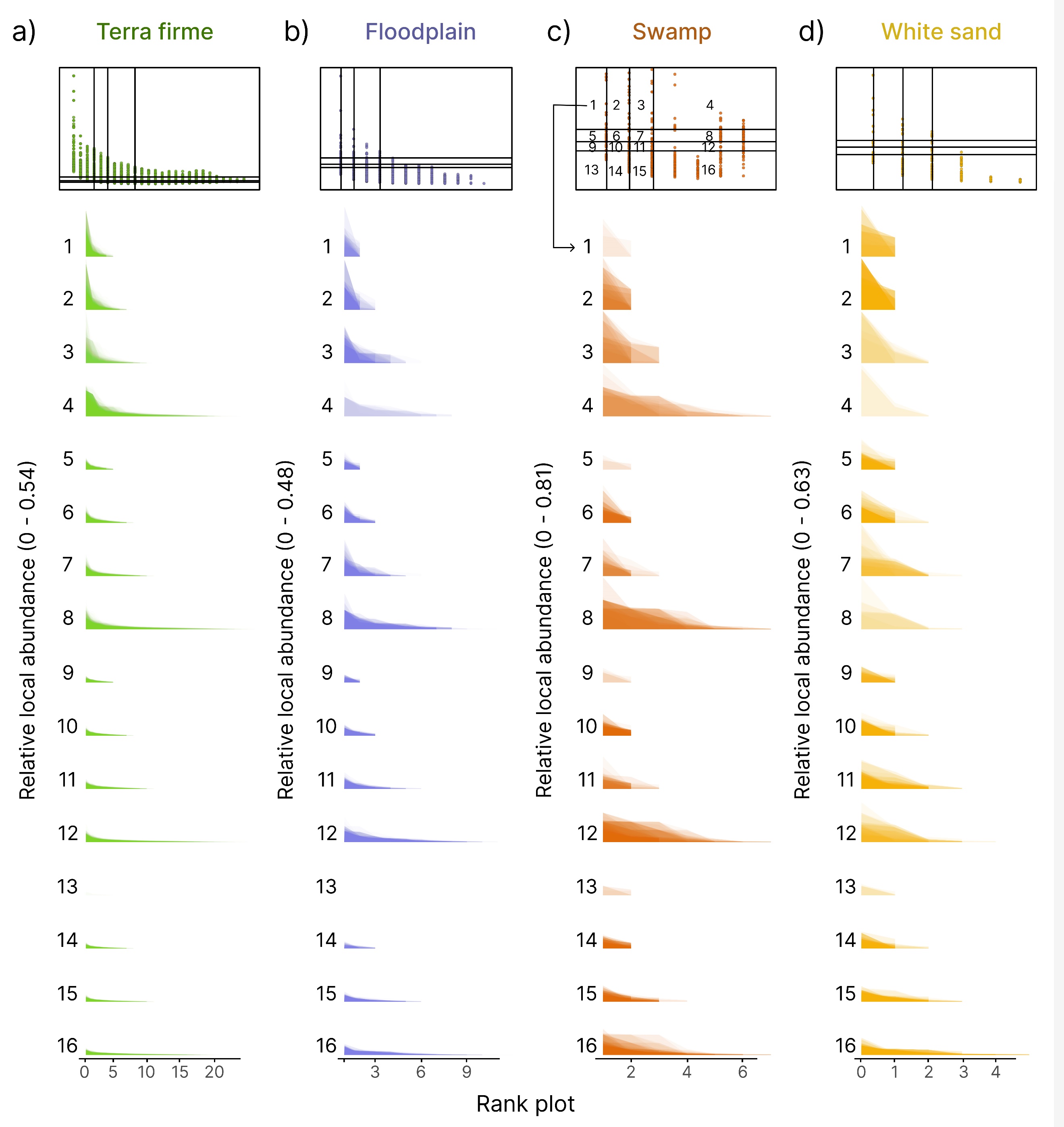


Figure S5. Species-level rank abundance distribution graphs to dominant species from 100 subsamples drawing one plot by 100 x 100 km square from the study area by habitat type: (a) *terra firme*, (b) floodplain, (c) swamp, and (d) white sand forests. Upper panels represent local abundance-regional frequency relationship of dominant species within each habitat type, with their quartiles. Numbers refer to each of the combination of quartiles of the two variables, local abundance and regional frequency.


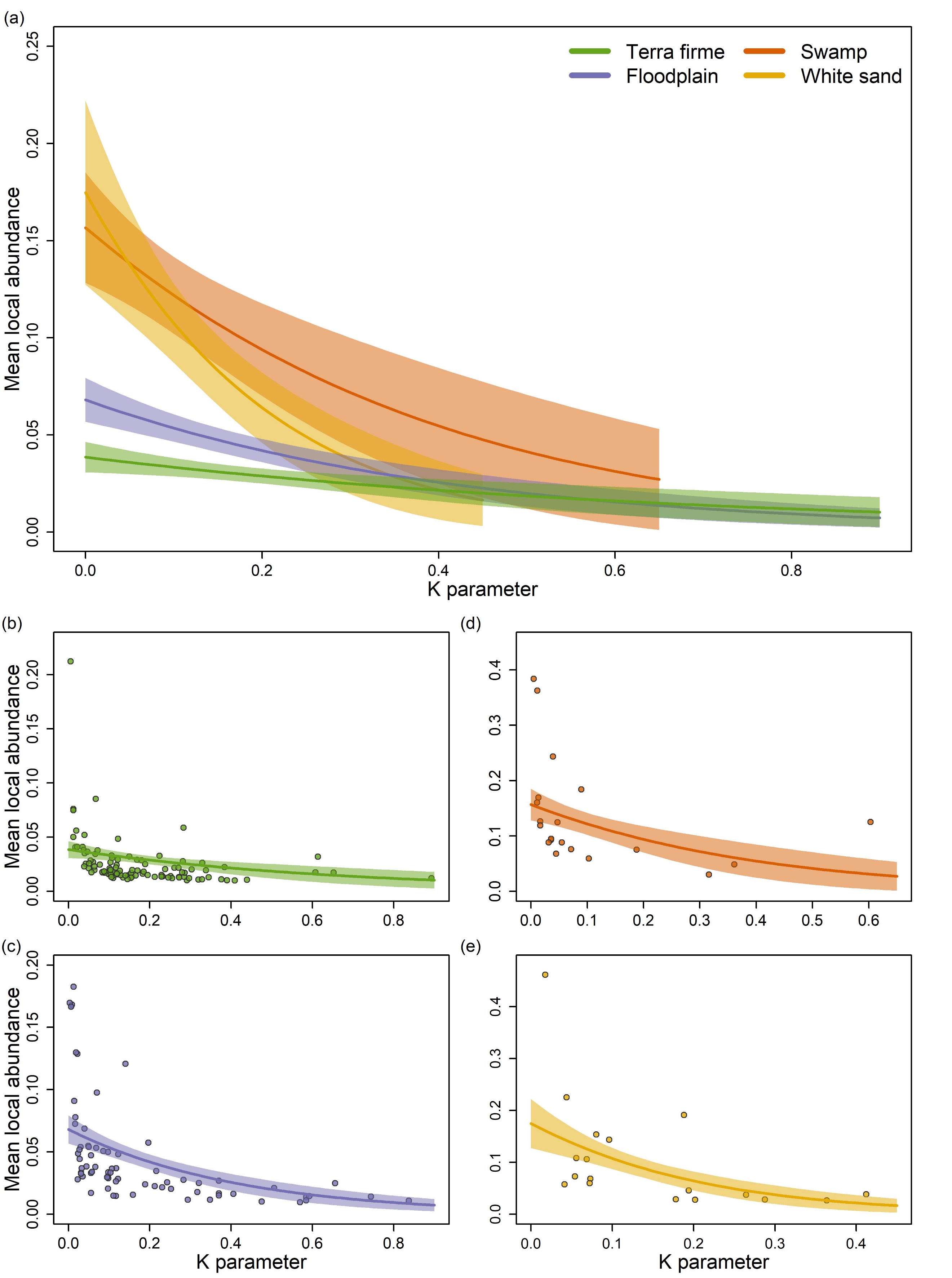


Figure S6. Model predictions for the best-fit beta regression model showing the relationship between the mean local abundance and the k parameter value of dominant species by habitat type. Lines represent mean generalized model fits, and shading represents 95% confidence intervals of model fits.


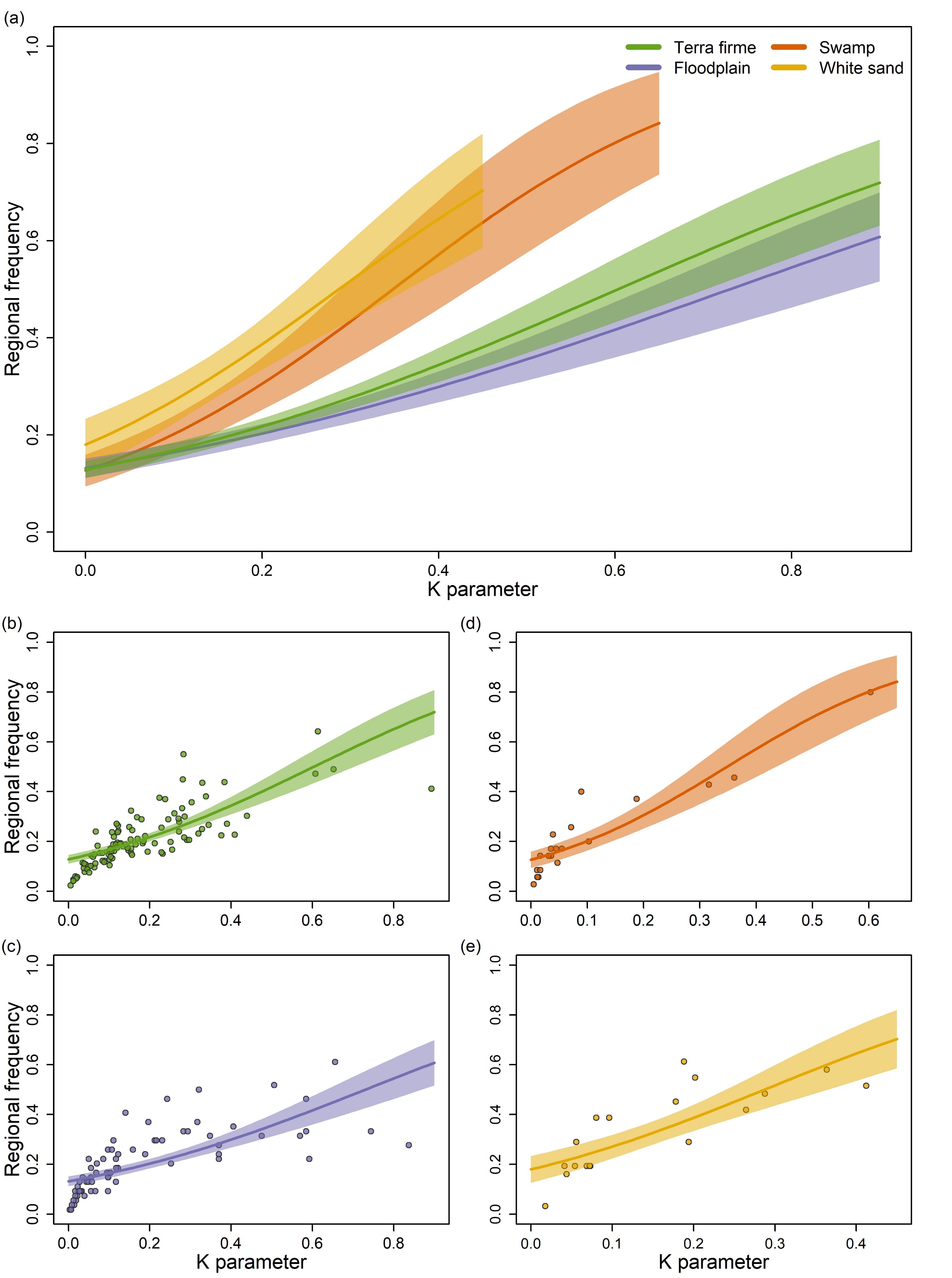


Figure S7. Model predictions for the best-fit beta regression model showing the relationship between the regional frequency and the k parameter value of dominant species by habitat type. Lines represent mean generalized model fits, and shading represents 95% confidence intervals of model fits.

**Literature cited**

Guisan, A. & Zimmermann, N.E. (2000). Predictive habitat distribution models in ecology. *Ecol. Modell.*, 135, 147–186. https://doi.org/10.1016/S0304-3800(00)00354-9

**APPENDIX S2: DOMINANT SPECIES INFORMATION**

**Table S6.** List of dominant species found in the four main habitat types of western Amazonia. Habitat type refers to the forest where the species was identified as dominant. ‘Cell’ refers to the combination of quartiles of local abundance and regional frequency where the species fell into. ‘Times’ refers to the number of times that each species was identified as ‘dominant’ in the 100 subsamples drawing one plot by 100 x 100 km square by habitat type. Representative examples of *local dominants*, *widespread dominants* and, *oligarchs* in each habitat type are labelled with superscript ^1,2,3^, respectively.

| **Species** | **Family** | **Mean local abundance** | **Regional frequency** | **k parameter** | **Habitat type** | **Cell** | **Times** |
| --- | --- | --- | --- | --- | --- | --- | --- |
| *Aspidosperma parvifolium* | Apocynaceae | 0.014 | 0.198 | 0.251 | *Terra firme* | 15 | 16 |
| *Astrocaryum murumuru* | Arecaceae | 0.048 | 0.264 | 0.122 | *Terra firme* | 4 | 100 |
| *Attalea racemosa*^1^ | Arecaceae | 0.212 | 0.023 | 0.005 | *Terra firme* | 1 | 16 |
| *Bactris concinna* | Arecaceae | 0.041 | 0.115 | 0.035 | *Terra firme* | 1 | 29 |
| *Bribria apiculata* | Violaceae | 0.076 | 0.047 | 0.012 | *Terra firme* | 1 | 22 |
| *Brosimum lactescens* | Moraceae | 0.010 | 0.227 | 0.409 | *Terra firme* | 15 | 100 |
| *Capparidastrum solum* | Capparaceae | 0.021 | 0.099 | 0.050 | *Terra firme* | 5 | 20 |
| *Carpotroche longifolia* | Achariaceae | 0.017 | 0.123 | 0.089 | *Terra firme* | 9 | 100 |
| *Cecropia sciadophylla* | Urticaceae | 0.012 | 0.188 | 0.110 | *Terra firme* | 14 | 59 |
| *Celtis schippii* | Cannabaceae | 0.022 | 0.313 | 0.261 | *Terra firme* | 8 | 100 |
| *Cheiloclinium cognatum* | Celastraceae | 0.013 | 0.180 | 0.135 | *Terra firme* | 14 | 12 |
| *Chrysochlamys ulei* | Clusiaceae | 0.041 | 0.060 | 0.017 | *Terra firme* | 1 | 30 |
| *Clarisia biflora* | Moraceae | 0.013 | 0.193 | 0.212 | *Terra firme* | 15 | 35 |
| *Clarisia racemosa* | Moraceae | 0.010 | 0.272 | 0.390 | *Terra firme* | 16 | 32 |
| *Conceveiba rhytidocarpa* | Euphorbiaceae | 0.011 | 0.225 | 0.376 | *Terra firme* | 15 | 58 |
| *Cordia nodosa* | Cordiaceae | 0.012 | 0.413 | 0.893 | *Terra firme* | 16 | 100 |
| *Dendropanax arboreus* | Araliaceae | 0.010 | 0.206 | 0.291 | *Terra firme* | 15 | 93 |
| *Drypetes amazonica* | Putranjivaceae | 0.017 | 0.243 | 0.123 | *Terra firme* | 11 | 69 |
| *Duguetia spixiana* | Annonaceae | 0.015 | 0.164 | 0.148 | *Terra firme* | 10 | 14 |
| *Eschweilera coriacea* | Lecythidaceae | 0.019 | 0.381 | 0.338 | *Terra firme* | 8 | 100 |
| *Euterpe precatoria* | Arecaceae | 0.022 | 0.439 | 0.384 | *Terra firme* | 8 | 100 |
| *Garcinia madruno* | Clusiaceae | 0.016 | 0.149 | 0.099 | *Terra firme* | 10 | 21 |
| *Guarea gomma* | Meliaceae | 0.013 | 0.217 | 0.285 | *Terra firme* | 15 | 20 |
| *Guarea kunthiana* | Meliaceae | 0.017 | 0.300 | 0.285 | *Terra firme* | 12 | 99 |
| *Guarea macrophylla* | Meliaceae | 0.021 | 0.371 | 0.238 | *Terra firme* | 8 | 99 |
| *Guarea pterorhachis* | Meliaceae | 0.017 | 0.123 | 0.084 | *Terra firme* | 9 | 52 |
| *Hasseltia floribunda* | Salicaceae | 0.016 | 0.219 | 0.112 | *Terra firme* | 11 | 59 |
| *Helicostylis tomentosa* | Moraceae | 0.012 | 0.209 | 0.153 | *Terra firme* | 15 | 81 |
| *Hevea guianensis* | Euphorbiaceae | 0.021 | 0.128 | 0.101 | *Terra firme* | 5 | 42 |
| *Hirtella excelsa* | Chrysobalanaceae | 0.013 | 0.154 | 0.154 | *Terra firme* | 14 | 1 |
| *Hirtella racemosa* | Chrysobalanaceae | 0.029 | 0.298 | 0.168 | *Terra firme* | 4 | 99 |
| *Hirtella triandra* | Chrysobalanaceae | 0.019 | 0.211 | 0.170 | *Terra firme* | 7 | 73 |
| *Inga ruiziana* | Fabaceae | 0.014 | 0.146 | 0.157 | *Terra firme* | 14 | 0 |
| *Iriartea deltoidea*^3^ | Arecaceae | 0.059 | 0.551 | 0.283 | *Terra firme* | 4 | 100 |
| *Iryanthera juruensis* | Myristicaceae | 0.026 | 0.436 | 0.329 | *Terra firme* | 8 | 99 |
| *Iryanthera laevis* | Myristicaceae | 0.018 | 0.334 | 0.279 | *Terra firme* | 12 | 100 |
| *Iryanthera paraensis* | Myristicaceae | 0.016 | 0.193 | 0.182 | *Terra firme* | 11 | 63 |
| *Ixora peruviana* | Rubiaceae | 0.020 | 0.097 | 0.064 | *Terra firme* | 5 | 7 |
| *Jacaranda copaia* | Bignoniaceae | 0.012 | 0.167 | 0.255 | *Terra firme* | 14 | 16 |
| *Lacistema aggregatum* | Lacistemataceae | 0.013 | 0.240 | 0.271 | *Terra firme* | 15 | 29 |
| *Leonia crassa* | Violaceae | 0.029 | 0.238 | 0.121 | *Terra firme* | 3 | 89 |
| *Leonia glycycarpa* | Violaceae | 0.017 | 0.491 | 0.652 | *Terra firme* | 12 | 100 |
| *Lepidocaryum tenue* | Arecaceae | 0.041 | 0.057 | 0.022 | *Terra firme* | 1 | 13 |
| *Lunania parviflora* | Salicaceae | 0.027 | 0.172 | 0.103 | *Terra firme* | 6 | 53 |
| *Mabea speciosa* | Euphorbiaceae | 0.035 | 0.078 | 0.040 | *Terra firme* | 1 | 47 |
| *Matisia malacocalyx* | Malvaceae | 0.015 | 0.162 | 0.112 | *Terra firme* | 10 | 74 |
| *Mollinedia killipii* | Monimiaceae | 0.025 | 0.154 | 0.077 | *Terra firme* | 6 | 61 |
| *Naucleopsis glabra* | Moraceae | 0.014 | 0.151 | 0.232 | *Terra firme* | 14 | 39 |
| *Nealchornea yapurensis* | Euphorbiaceae | 0.015 | 0.159 | 0.195 | *Terra firme* | 10 | 44 |
| *Neea macrophylla* | Nyctaginaceae | 0.015 | 0.188 | 0.165 | *Terra firme* | 10 | 5 |
| *Oenocarpus bataua* | Arecaceae | 0.024 | 0.272 | 0.118 | *Terra firme* | 8 | 100 |
| *Oenocarpus mapora* | Arecaceae | 0.029 | 0.290 | 0.180 | *Terra firme* | 4 | 97 |
| *Otoba glycycarpa* | Myristicaceae | 0.035 | 0.112 | 0.066 | *Terra firme* | 1 | 38 |
| *Otoba parvifolia* | Myristicaceae | 0.032 | 0.258 | 0.150 | *Terra firme* | 4 | 100 |
| *Oxandra mediocris* | Annonaceae | 0.018 | 0.144 | 0.056 | *Terra firme* | 10 | 61 |
| *Pausandra trianae* | Euphorbiaceae | 0.052 | 0.107 | 0.039 | *Terra firme* | 1 | 100 |
| *Piper obliquum* | Piperaceae | 0.018 | 0.154 | 0.083 | *Terra firme* | 10 | 37 |
| *Piper pseudoarboreum* | Piperaceae | 0.027 | 0.138 | 0.060 | *Terra firme* | 5 | 46 |
| *Piper reticulatum* | Piperaceae | 0.026 | 0.107 | 0.043 | *Terra firme* | 5 | 24 |
| *Poulsenia armata* | Moraceae | 0.033 | 0.170 | 0.106 | *Terra firme* | 2 | 75 |
| *Pourouma cecropiifolia* | Urticaceae | 0.013 | 0.266 | 0.345 | *Terra firme* | 16 | 100 |
| *Pourouma guianensis* | Urticaceae | 0.016 | 0.196 | 0.120 | *Terra firme* | 11 | 65 |
| *Pourouma minor* | Urticaceae | 0.020 | 0.358 | 0.303 | *Terra firme* | 8 | 100 |
| *Pouteria torta* | Sapotaceae | 0.011 | 0.303 | 0.439 | *Terra firme* | 16 | 24 |
| *Protium amazonicum* | Burseraceae | 0.018 | 0.201 | 0.185 | *Terra firme* | 11 | 99 |
| *Protium apiculatum* | Burseraceae | 0.018 | 0.117 | 0.088 | *Terra firme* | 5 | 28 |
| *Protium nodulosum* | Burseraceae | 0.018 | 0.159 | 0.101 | *Terra firme* | 10 | 55 |
| *Protium stevensonii* | Burseraceae | 0.018 | 0.133 | 0.108 | *Terra firme* | 9 | 13 |
| *Protium tenuifolium* | Burseraceae | 0.022 | 0.188 | 0.117 | *Terra firme* | 6 | 38 |
| *Pseudolmedia laevigata*^2^ | Moraceae | 0.017 | 0.324 | 0.154 | *Terra firme* | 12 | 100 |
| *Pseudolmedia laevis* | Moraceae | 0.032 | 0.642 | 0.613 | *Terra firme* | 4 | 100 |
| *Pseudolmedia macrophylla* | Moraceae | 0.011 | 0.232 | 0.320 | *Terra firme* | 15 | 37 |
| *Pseudosenefeldera inclinata* | Euphorbiaceae | 0.038 | 0.094 | 0.038 | *Terra firme* | 1 | 61 |
| *Quararibea wittii* | Malvaceae | 0.038 | 0.183 | 0.072 | *Terra firme* | 2 | 100 |
| *Rinorea lindeniana* | Violaceae | 0.029 | 0.102 | 0.054 | *Terra firme* | 1 | 81 |
| *Rinorea pubiflora* | Violaceae | 0.050 | 0.042 | 0.012 | *Terra firme* | 1 | 11 |
| *Rinorea racemosa* | Violaceae | 0.037 | 0.089 | 0.046 | *Terra firme* | 1 | 31 |
| *Rinorea viridifolia* | Violaceae | 0.085 | 0.240 | 0.067 | *Terra firme* | 3 | 99 |
| *Rinoreocarpus ulei* | Violaceae | 0.019 | 0.104 | 0.101 | *Terra firme* | 5 | 14 |
| *Roucheria columbiana* | Linaceae | 0.012 | 0.172 | 0.154 | *Terra firme* | 14 | 62 |
| *Ruizodendron ovale* | Annonaceae | 0.015 | 0.193 | 0.128 | *Terra firme* | 11 | 36 |
| *Siparuna bifida* | Siparunaceae | 0.023 | 0.149 | 0.064 | *Terra firme* | 6 | 27 |
| *Siparuna cuspidata* | Siparunaceae | 0.031 | 0.238 | 0.107 | *Terra firme* | 3 | 80 |
| *Siparuna decipiens* | Siparunaceae | 0.033 | 0.376 | 0.224 | *Terra firme* | 4 | 100 |
| *Siparuna guianensis* | Siparunaceae | 0.023 | 0.112 | 0.039 | *Terra firme* | 5 | 29 |
| *Socratea exorrhiza* | Arecaceae | 0.028 | 0.449 | 0.281 | *Terra firme* | 4 | 100 |
| *Sorocea briquetii* | Moraceae | 0.013 | 0.290 | 0.245 | *Terra firme* | 16 | 48 |
| *Sorocea pubivena* | Moraceae | 0.015 | 0.157 | 0.228 | *Terra firme* | 10 | 60 |
| *Styloceras brokawii* | Buxaceae | 0.056 | 0.052 | 0.019 | *Terra firme* | 1 | 23 |
| *Stylogyne ardisioides* | Primulaceae | 0.015 | 0.138 | 0.104 | *Terra firme* | 9 | 9 |
| *Symphonia globulifera* | Clusiaceae | 0.013 | 0.279 | 0.272 | *Terra firme* | 16 | 30 |
| *Tachigali bracteosa* | Fabaceae | 0.019 | 0.191 | 0.126 | *Terra firme* | 7 | 41 |
| *Tachigali poeppigiana* | Fabaceae | 0.016 | 0.175 | 0.119 | *Terra firme* | 10 | 26 |
| *Tapirira guianensis* | Anacardiaceae | 0.011 | 0.251 | 0.329 | *Terra firme* | 15 | 100 |
| *Tapura juruana* | Dichapetalaceae | 0.011 | 0.175 | 0.145 | *Terra firme* | 14 | 6 |
| *Theobroma cacao* | Malvaceae | 0.020 | 0.245 | 0.157 | *Terra firme* | 7 | 88 |
| *Theobroma subincanum* | Malvaceae | 0.012 | 0.206 | 0.297 | *Terra firme* | 15 | 100 |
| *Trichilia pleeana* | Meliaceae | 0.015 | 0.188 | 0.140 | *Terra firme* | 10 | 31 |
| *Trichilia quadrijuga* | Meliaceae | 0.013 | 0.162 | 0.105 | *Terra firme* | 14 | 19 |
| *Unonopsis floribunda* | Annonaceae | 0.022 | 0.292 | 0.270 | *Terra firme* | 8 | 95 |
| *Virola calophylla* | Myristicaceae | 0.018 | 0.473 | 0.608 | *Terra firme* | 12 | 100 |
| *Virola elongata* | Myristicaceae | 0.020 | 0.170 | 0.103 | *Terra firme* | 6 | 99 |
| *Virola pavonis* | Myristicaceae | 0.025 | 0.222 | 0.189 | *Terra firme* | 7 | 97 |
| *Virola sebifera* | Myristicaceae | 0.014 | 0.256 | 0.229 | *Terra firme* | 15 | 63 |
| *Wettinia augusta* | Arecaceae | 0.075 | 0.042 | 0.012 | *Terra firme* | 1 | 64 |
| *Zygia basijuga* | Fabaceae | 0.026 | 0.076 | 0.051 | *Terra firme* | 5 | 36 |
| *Astrocaryum murumuru* | Arecaceae | 0.017 | 0.278 | 0.370 | Floodplain | 15 | 39 |
| *Batocarpus amazonicus* | Moraceae | 0.050 | 0.093 | 0.097 | Floodplain | 5 | 12 |
| *Caraipa valioi* | Calophyllaceae | 0.167 | 0.019 | 0.006 | Floodplain | 1 | 30 |
| *Casearia sylvestris* | Salicaceae | 0.047 | 0.093 | 0.055 | Floodplain | 5 | 13 |
| *Cecropia membranacea* | Urticaceae | 0.049 | 0.093 | 0.024 | Floodplain | 5 | 42 |
| *Celtis schippii* | Cannabaceae | 0.014 | 0.333 | 0.584 | Floodplain | 16 | 59 |
| *Clarisia biflora* | Moraceae | 0.012 | 0.315 | 0.348 | Floodplain | 16 | 6 |
| *Coccoloba densifrons* | Polygonaceae | 0.022 | 0.296 | 0.230 | Floodplain | 11 | 54 |
| *Cordia nodosa* | Cordiaceae | 0.011 | 0.463 | 0.584 | Floodplain | 16 | 98 |
| *Coussarea brevicaulis* | Rubiaceae | 0.051 | 0.222 | 0.086 | Floodplain | 7 | 68 |
| *Coussarea hirticalyx* | Rubiaceae | 0.129 | 0.074 | 0.022 | Floodplain | 1 | 37 |
| *Coussarea macrophylla* | Rubiaceae | 0.073 | 0.093 | 0.016 | Floodplain | 1 | 40 |
| *Drypetes amazonica* | Putranjivaceae | 0.026 | 0.463 | 0.243 | Floodplain | 12 | 100 |
| *Eschweilera albiflora* | Lecythidaceae | 0.027 | 0.222 | 0.370 | Floodplain | 11 | 35 |
| *Eschweilera parvifolia* | Lecythidaceae | 0.033 | 0.148 | 0.055 | Floodplain | 6 | 94 |
| *Euterpe precatoria* | Arecaceae | 0.021 | 0.519 | 0.506 | Floodplain | 12 | 97 |
| *Grias peruviana* | Lecythidaceae | 0.028 | 0.185 | 0.121 | Floodplain | 10 | 14 |
| *Guarea guidonia* | Meliaceae | 0.030 | 0.148 | 0.035 | Floodplain | 10 | 42 |
| *Guarea kunthiana* | Meliaceae | 0.031 | 0.259 | 0.097 | Floodplain | 11 | 48 |
| *Guarea macrophylla*^2^ | Meliaceae | 0.025 | 0.611 | 0.656 | Floodplain | 12 | 100 |
| *Guarea pterorhachis* | Meliaceae | 0.037 | 0.185 | 0.117 | Floodplain | 6 | 40 |
| *Gustavia augusta* | Lecythidaceae | 0.130 | 0.056 | 0.018 | Floodplain | 1 | 100 |
| *Hasseltia floribunda* | Salicaceae | 0.010 | 0.315 | 0.570 | Floodplain | 16 | 23 |
| *Hieronyma alchorneoides* | Phyllanthaceae | 0.011 | 0.278 | 0.837 | Floodplain | 15 | 7 |
| *Himatanthus sucuuba* | Apocynaceae | 0.020 | 0.204 | 0.252 | Floodplain | 15 | 56 |
| *Hirtella racemosa* | Chrysobalanaceae | 0.078 | 0.074 | 0.017 | Floodplain | 1 | 38 |
| *Hydrochorea corymbosa* | Fabaceae | 0.091 | 0.037 | 0.014 | Floodplain | 1 | 28 |
| *Iriartea deltoidea* | Arecaceae | 0.057 | 0.370 | 0.197 | Floodplain | 4 | 100 |
| *Iryanthera tessmannii* | Myristicaceae | 0.038 | 0.093 | 0.066 | Floodplain | 5 | 21 |
| *Laetia corymbulosa* | Salicaceae | 0.183 | 0.056 | 0.012 | Floodplain | 1 | 79 |
| *Leonia crassa* | Violaceae | 0.015 | 0.222 | 0.116 | Floodplain | 15 | 16 |
| *Leonia glycycarpa* | Violaceae | 0.028 | 0.333 | 0.283 | Floodplain | 12 | 100 |
| *Luehea seemannii*^1^ | Malvaceae | 0.168 | 0.037 | 0.008 | Floodplain | 1 | 58 |
| *Lunania parviflora* | Salicaceae | 0.029 | 0.148 | 0.098 | Floodplain | 10 | 100 |
| *Mabea trianae* | Euphorbiaceae | 0.170 | 0.019 | 0.003 | Floodplain | 1 | 100 |
| *Maquira coriacea* | Moraceae | 0.024 | 0.241 | 0.188 | Floodplain | 11 | 24 |
| *Margaritaria nobilis* | Phyllanthaceae | 0.017 | 0.185 | 0.055 | Floodplain | 14 | 22 |
| *Matisia bracteolosa* | Malvaceae | 0.038 | 0.130 | 0.044 | Floodplain | 6 | 29 |
| *Mauritia flexuosa* | Arecaceae | 0.069 | 0.074 | 0.039 | Floodplain | 1 | 30 |
| *Micropholis venulosa* | Sapotaceae | 0.033 | 0.093 | 0.031 | Floodplain | 5 | 7 |
| *Mollinedia ovata* | Monimiaceae | 0.055 | 0.130 | 0.049 | Floodplain | 2 | 27 |
| *Neea floribunda* | Nyctaginaceae | 0.026 | 0.130 | 0.116 | Floodplain | 10 | 25 |
| *Oenocarpus bataua* | Arecaceae | 0.044 | 0.130 | 0.027 | Floodplain | 6 | 70 |
| *Otoba parvifolia*^3^ | Myristicaceae | 0.121 | 0.407 | 0.140 | Floodplain | 4 | 89 |
| *Oxandra mediocris* | Annonaceae | 0.015 | 0.241 | 0.370 | Floodplain | 15 | 82 |
| *Pausandra trianae* | Euphorbiaceae | 0.037 | 0.093 | 0.032 | Floodplain | 5 | 48 |
| *Pentagonia spathicalyx* | Rubiaceae | 0.053 | 0.167 | 0.068 | Floodplain | 2 | 18 |
| *Perebea longepedunculata* | Moraceae | 0.054 | 0.093 | 0.030 | Floodplain | 1 | 32 |
| *Perebea xanthochyma* | Moraceae | 0.023 | 0.296 | 0.212 | Floodplain | 11 | 37 |
| *Phytelephas tenuicaulis* | Arecaceae | 0.048 | 0.241 | 0.122 | Floodplain | 7 | 69 |
| *Piper crassinervium* | Piperaceae | 0.028 | 0.111 | 0.023 | Floodplain | 10 | 9 |
| *Pourouma cecropiifolia* | Urticaceae | 0.015 | 0.296 | 0.110 | Floodplain | 15 | 39 |
| *Pouteria procera* | Sapotaceae | 0.032 | 0.093 | 0.031 | Floodplain | 9 | 10 |
| *Pouteria torta* | Sapotaceae | 0.010 | 0.315 | 0.475 | Floodplain | 16 | 15 |
| *Pseudolmedia laevis* | Moraceae | 0.035 | 0.296 | 0.216 | Floodplain | 7 | 100 |
| *Quararibea wittii* | Malvaceae | 0.036 | 0.259 | 0.107 | Floodplain | 7 | 100 |
| *Rinorea lindeniana* | Violaceae | 0.052 | 0.093 | 0.027 | Floodplain | 5 | 44 |
| *Rinorea viridifolia* | Violaceae | 0.098 | 0.204 | 0.070 | Floodplain | 3 | 100 |
| *Sacoglottis ceratocarpa* | Humiriaceae | 0.167 | 0.019 | 0.006 | Floodplain | 1 | 30 |
| *Sapium marmieri* | Euphorbiaceae | 0.016 | 0.259 | 0.158 | Floodplain | 15 | 61 |
| *Siparuna decipiens* | Siparunaceae | 0.034 | 0.130 | 0.057 | Floodplain | 6 | 12 |
| *Socratea exorrhiza* | Arecaceae | 0.025 | 0.500 | 0.320 | Floodplain | 12 | 100 |
| *Sorocea briquetii* | Moraceae | 0.015 | 0.222 | 0.592 | Floodplain | 15 | 53 |
| *Sorocea steinbachii* | Moraceae | 0.054 | 0.222 | 0.050 | Floodplain | 3 | 66 |
| *Spondias mombin* | Anacardiaceae | 0.009 | 0.370 | 8.211E+14 | Floodplain | 16 | 8 |
| *Stylogyne ardisioides* | Primulaceae | 0.021 | 0.148 | 0.097 | Floodplain | 10 | 24 |
| *Theobroma cacao* | Malvaceae | 0.018 | 0.370 | 0.317 | Floodplain | 16 | 77 |
| *Triplaris americana* | Polygonaceae | 0.014 | 0.333 | 0.744 | Floodplain | 16 | 30 |
| *Unonopsis floribunda* | Annonaceae | 0.016 | 0.352 | 0.406 | Floodplain | 16 | 77 |
| *Virola calophylla* | Myristicaceae | 0.012 | 0.333 | 0.293 | Floodplain | 16 | 37 |
| *Virola elongata* | Myristicaceae | 0.034 | 0.167 | 0.098 | Floodplain | 6 | 24 |
| *Virola pavonis* | Myristicaceae | 0.030 | 0.167 | 0.095 | Floodplain | 10 | 34 |
| *Zygia cataractae* | Fabaceae | 0.033 | 0.167 | 0.103 | Floodplain | 6 | 45 |
| *Attalea butyracea* | Arecaceae | 0.125 | 0.114 | 0.047 | Swamp | 6 | 100 |
| *Calophyllum brasiliense* | Calophyllaceae | 0.169 | 0.057 | 0.013 | Swamp | 1 | 54 |
| *Cespedesia spathulata* | Ochnaceae | 0.068 | 0.171 | 0.045 | Swamp | 14 | 43 |
| *Euterpe catinga* | Arecaceae | 0.363 | 0.057 | 0.011 | Swamp | 1 | 100 |
| *Euterpe precatoria* | Arecaceae | 0.075 | 0.371 | 0.188 | Swamp | 16 | 85 |
| *Graffenrieda limbata*^1^ | Melastomataceae | 0.384 | 0.029 | 0.005 | Swamp | 1 | 28 |
| *Hevea guianensis* | Euphorbiaceae | 0.076 | 0.257 | 0.071 | Swamp | 11 | 31 |
| *Ilex laureola* | Aquifoliaceae | 0.160 | 0.086 | 0.011 | Swamp | 5 | 12 |
| *Lueheopsis hoehnei* | Malvaceae | 0.093 | 0.171 | 0.035 | Swamp | 10 | 34 |
| *Macrolobium angustifolium* | Fabaceae | 0.059 | 0.200 | 0.103 | Swamp | 15 | 33 |
| *Macrolobium multijugum* | Fabaceae | 0.126 | 0.143 | 0.016 | Swamp | 6 | 8 |
| *Mauritia flexuosa*^3^ | Arecaceae | 0.125 | 0.800 | 0.603 | Swamp | 8 | 100 |
| *Mauritiella armata* | Arecaceae | 0.088 | 0.171 | 0.055 | Swamp | 10 | 51 |
| *Pachira brevipes* | Malvaceae | 0.244 | 0.229 | 0.039 | Swamp | 3 | 75 |
| *Pagamea guianensis* | Rubiaceae | 0.119 | 0.086 | 0.016 | Swamp | 5 | 12 |
| *Platycarpum loretense* | Rubiaceae | 0.095 | 0.143 | 0.036 | Swamp | 10 | 53 |
| *Symphonia globulifera*^2^ | Clusiaceae | 0.030 | 0.429 | 0.316 | Swamp | 16 | 21 |
| *Tabebuia insignis* | Bignoniaceae | 0.184 | 0.400 | 0.089 | Swamp | 4 | 69 |
| *Triplaris weigeltiana* | Polygonaceae | 0.089 | 0.143 | 0.031 | Swamp | 10 | 10 |
| *Virola pavonis* | Myristicaceae | 0.049 | 0.457 | 0.361 | Swamp | 16 | 45 |
| *Anaxagorea manausensis* | Annonaceae | 0.073 | 0.194 | 0.054 | White sand | 5 | 5 |
| *Calophyllum brasiliense* | Calophyllaceae | 0.058 | 0.194 | 0.041 | White sand | 9 | 32 |
| *Caraipa tereticaulis* | Calophyllaceae | 0.108 | 0.290 | 0.056 | White sand | 6 | 76 |
| *Caraipa utilis* | Calophyllaceae | 0.154 | 0.387 | 0.080 | White sand | 3 | 80 |
| *Chrysophyllum sanguinolentum* | Sapotaceae | 0.029 | 0.452 | 0.178 | White sand | 15 | 94 |
| *Dendropanax umbellatus* | Araliaceae | 0.037 | 0.419 | 0.264 | White sand | 15 | 0 |
| *Dicymbe uaiparuensis* | Fabaceae | 0.144 | 0.387 | 0.096 | White sand | 3 | 39 |
| *Euterpe catinga* | Arecaceae | 0.106 | 0.194 | 0.068 | White sand | 5 | 77 |
| *Haploclathra cordata* | Calophyllaceae | 0.225 | 0.161 | 0.044 | White sand | 1 | 80 |
| *Macrolobium limbatum* | Fabaceae | 0.039 | 0.516 | 0.412 | White sand | 12 | 13 |
| *Macrolobium microcalyx*^2^ | Fabaceae | 0.027 | 0.581 | 0.364 | White sand | 16 | 7 |
| *Matayba inelegans* | Sapindaceae | 0.028 | 0.548 | 0.202 | White sand | 16 | 64 |
| *Neocouma ternstroemiacea*^1^ | Apocynaceae | 0.462 | 0.032 | 0.017 | White sand | 1 | 100 |
| *Oxandra euneura* | Annonaceae | 0.068 | 0.194 | 0.073 | White sand | 5 | 10 |
| *Pachira brevipes*^3^ | Malvaceae | 0.191 | 0.613 | 0.188 | White sand | 4 | 88 |
| *Parkia igneiflora* | Fabaceae | 0.028 | 0.484 | 0.288 | White sand | 16 | 91 |
| *Sloanea spathulata* | Elaeocarpaceae | 0.060 | 0.194 | 0.072 | White sand | 9 | 2 |
| *Tachigali paniculata* | Fabaceae | 0.046 | 0.290 | 0.194 | White sand | 10 | 52 |
